# When a knockout is an Achilles’ heel: Resistance to one potyvirus species triggers hypersusceptibility to another one in *Arabidopsis thaliana*

**DOI:** 10.1111/mpp.13031

**Published:** 2020-12-29

**Authors:** Delyan Zafirov, Nathalie Giovinazzo, Anna Bastet, Jean‐Luc Gallois

**Affiliations:** ^1^ GAFL INRAE Montfavet France

**Keywords:** *Arabidopsis thaliana*, CRISPR, eIF4E, genetic resistance, potyvirus, susceptibility, turnip mosaic virus

## Abstract

The translation initiation factors 4E are a small family of major susceptibility factors to potyviruses. It has been suggested that knocking out these genes could provide genetic resistance in crops when natural resistance alleles, which encode functional eIF4E proteins, are not available. Here, using the well‐characterized *Arabidopsis thaliana–*potyvirus pathosystem, we evaluate the resistance spectrum of plants knocked out for *eIF4E1*, the susceptibility factor to clover yellow vein virus (ClYVV). We show that besides resistance to ClYVV, the *eIF4E1* loss of function is associated with hypersusceptibility to turnip mosaic virus (TuMV), a potyvirus known to rely on the paralog host factor *eIFiso4E*. On TuMV infection, plants knocked out for *eIF4E1* display striking developmental defects such as early senescence and primordia development stoppage. This phenotype is coupled with a strong TuMV overaccumulation throughout the plant, while remarkably the levels of the viral target eIFiso4E remain uninfluenced. Our data suggest that this hypersusceptibility cannot be explained by virus evolution leading to a gain of TuMV aggressiveness. Furthermore, we report that a functional *eIF4E1* resistance allele engineered by CRISPR/Cas9 base‐editing technology successfully circumvents the increase of TuMV susceptibility conditioned by *eIF4E1* disruption. These findings in *Arabidopsis* add to several previous findings in crops suggesting that resistance based on knocking out *eIF4E* factors should be avoided in plant breeding, as it could also expose the plant to the severe threat of potyviruses able to recruit alternative *eIF4E* copies. At the same time, it provides a simple model that can help understanding of the homeostasis among eIF4E proteins in the plant cell and what makes them available to potyviruses.

## Graphical abstract

Knocking out the susceptibility factor eIF4E1 to generate resistance to a potyvirus triggers hypersusceptibility to another potyvirus that targets the paralog eIFiso4E; a CRISPR functional allele does not.

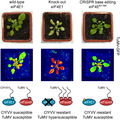

## 1 INTRODUCTION

Plant pathogens are responsible for significant agricultural and economic losses worldwide (Li et al., 2020). Genetic resistance is an important trait looked for in crop improvement programmes as it provides an effective and environmentally safe strategy to control pathogens in fields. One important challenge of breeding is to be able to translate known resistance mechanisms from species where they have been characterized to species where they have not yet been. This is particularly important for orphan crops to speed up breeding and make up for the lack of investment (Jacob et al., 2018). Dominant genetic resistances based on *R* genes, relying on response to the detection of pathogen avirulence factors, are very well developed and can sometimes be transferred to other species (Lacombe et al., 2010; for review Rodriguez‐Moreno et al., 2017), but often with limited success (van Wersch et al., 2020). On the contrary, it is expected that resistances by loss of susceptibility, based on the absence or incompatibility of host susceptibility factors required for pathogen proliferation, are possibly easier to translate among crops as they are supposed to target conserved mechanisms. For example, resistance to powdery mildew associated with *Mildew resistance locus o* (*Mlo)* genes characterized in barley (*Hordeum vulgare*) can be set up in tomato (*Solanum lycopersicum*) or apple (*Malus domestica*) (Kusch & Panstruga, 2017). Resistance to *Xanthomonas* species obtained by regulating the expression of the glucose transporter *SWEET* genes reported in rice (*Oryza sativa*) (Oliva et al., 2019) is conserved in cassava (*Manihot esculenta*) (Cohn et al., 2014) and cotton (*Gossypium* spp.) (Cox et al., 2017). Finally, the role of translation initiation factors *eIF4E* in susceptibility to many single‐stranded positive‐sense RNA (ssRNA+) viruses in crops has been shown to extend to caliciviruses affecting animals and humans (Hosmillo et al., 2014; Robaglia & Caranta, 2006). However, translating resistance mechanisms by loss of susceptibility can be hindered by gene redundancy among susceptibility factors belonging to small multigene families. Susceptibility factors having overlapping functions could offer diversified baits to the pathogen to hijack, and thus provide new routes of susceptibility or resistance‐breaking opportunities. This is exemplified by resistance associated with translation initiation factors eIF4E to potyviruses in crops (Gallois et al., 2018).

Translation initiation factors *eIF4E* and their paralogous counterparts *eIFiso4E* are a major source of resistance to members of the *Potyviridae* family as well as related ssRNA+ viruses and constitute traits of great agronomical importance (Robaglia & Caranta, 2006; Wang & Krishnaswamy, 2012). Since their discovery in pepper (*Capsicum annuum*) 20 years ago (Ruffel et al., 2002), they have become a model for the implementation of resistance through both classical breeding and biotechnology, using allele mining, and diverse ways of gene inactivation (RNAi, TILLING, CRISPR) (Schmitt‐Keichinger, 2019). Natural *eIF4E* resistance alleles are characterized in many crops by the presence of a small set of nonsynonymous mutations associated with amino acid changes in specific regions of the eIF4E protein. As a result, the *eIF4E* resistance alleles encode proteins that are still functional in their translation initiation function, but can no longer be recruited by the virus, leading to resistance. However, such resistance alleles are not present in the natural variability of some crops. For instance, only dominant resistance genes have been characterized in potato (*Solanum tuberosum*) in which infection by potato virus Y (PVY) induces a strong tuber necrosis phenotype (Glais et al., 2015). A similar situation occurs in other economically important crops such as papaya (*Carica papaya*) and cassava challenged by papaya ringspot virus (PRSV) and cassava brown streak virus (CBSV), respectively (Bart & Taylor, 2017; Gonsalves, 1998). When functional *eIF4E* resistance alleles are not available, biotechnological approaches to transfer resistance can be carried out by knocking down or out the susceptibility factors. Although associated in some cases with efficient resistance, the resulting plants could be affected in their development because of the important physiological role of eIF4E, and be prone to limited resistance spectrum or to resistance breaking because of gene redundancy (Bastet et al., 2017). Overall, we posit that, taking opportunity of the genome editing tools associated with CRISPR‐Cas9, a good strategy would be to copy in *eIF4E* mutations that have been selected by natural variation rather than knocking out the gene. Indeed, using *Arabidopsis thaliana* as a model, we showed that such mutations could be copied across species, namely from pea (*Pisum sativum*) *eIF4E* resistance alleles to the *Arabidopsis* *eIF4E1*, effectively transferring resistance (Bastet et al., 2018). We further showed that the more relevant mutations could be inserted in transgene‐free plants by genome base editing, allowing resistance at no yield cost (Bastet et al., 2019).

Because potyviruses are known to selectively require different *eIF4E* paralogs to establish infection (*eIF4E*, *eIFiso4E*, or the atypical *nCBP* factor) (Duprat et al., 2002; Gomez et al., 2019; Nicaise et al., 2007; Sato et al., 2005), we reasoned that reshuffling this selectivity by inactivating one paralog could affect global plant susceptibility. In the present study, we took advantage of the *A. thaliana–*potyvirus pathosystem where clover yellow vein virus (ClYVV) recruits eIF4E1 while turnip mosaic virus (TuMV) uses the counterpart eIFiso4E to accomplish their infection cycles. We show that knocking out *eIF4E1*, to generate resistance to ClYVV, is at the same time responsible for a remarkable increase in susceptibility towards TuMV. This aggravated phenotype to TuMV infection is associated with an increase in virus load throughout the plant and can be averted by the deployment of a functional *eIF4E1* resistance allele. We propose that because potyviruses specifically hijack different eIF4E factors, the strategy of developing resistance by *eIF4E* loss of function could be jeopardized by existing or emerging viruses able to recruit the remaining paralogs in the plant.

## 2 RESULTS

### 2.1 Loss of function in *eIF4E1* is associated with an increased severity of symptoms on infection by TuMV

**FIGURE 1:**
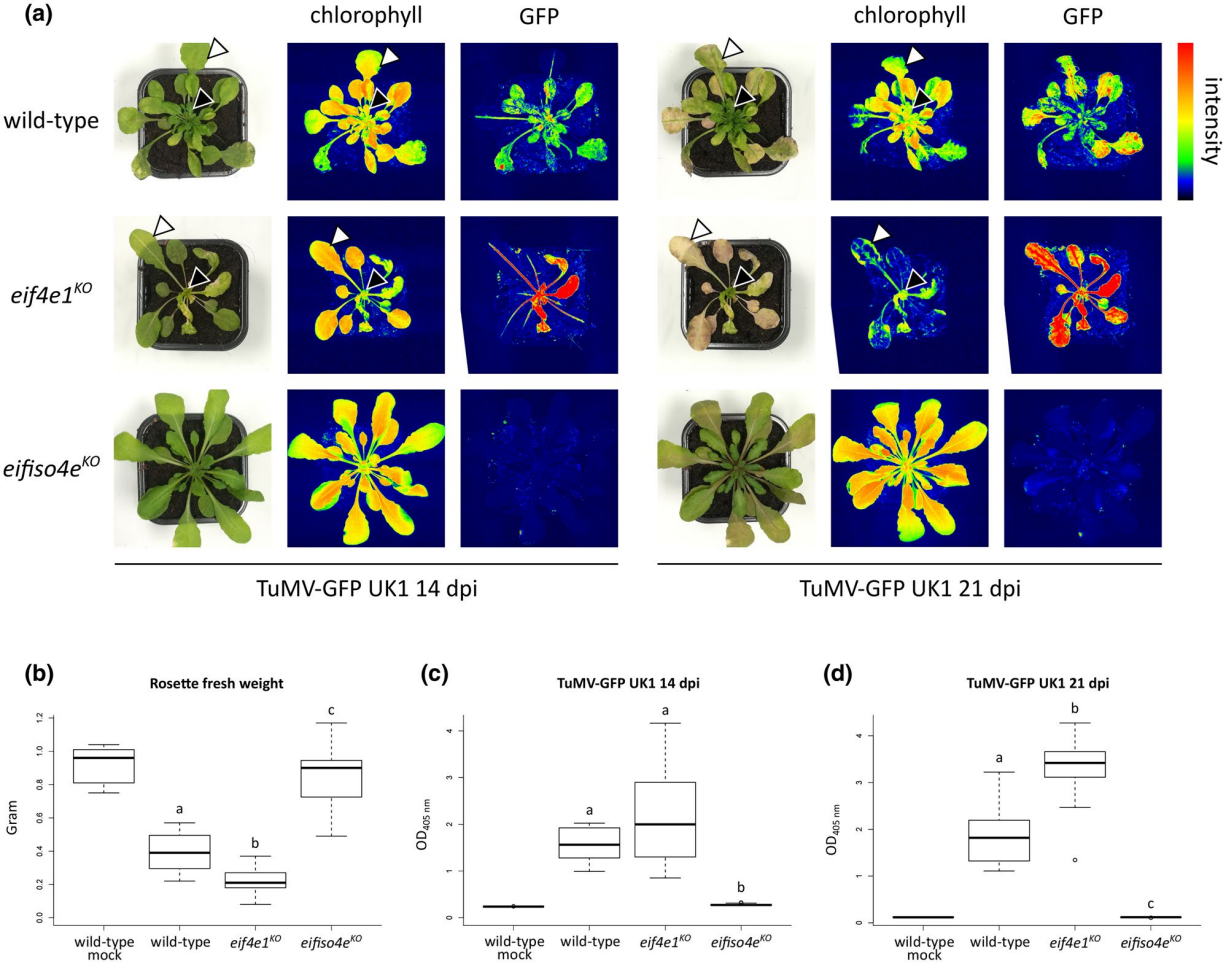
The *Arabidopsis* *eif4e1^KO^* mutant displays an enhanced susceptibility towards the UK1 isolate of turnip mosaic virus (TuMV). (a) Phenotypic comparison of representative plants on TuMV‐GFP UK1 infection at 14 and 21 days postinoculation (dpi). Photographs were taken under natural light conditions (left panel) and under wavelengths specific for chlorophyll excitation (middle panel) or green fluorescent protein (GFP) excitation (right panel) by using GFP camera (PSI) fluorescence imaging. Fluorescence is represented by false colours ranging from blue (low intensity) to red (high intensity). White and black arrowheads represent leaves formed prior to and after the viral inoculation step, respectively. The same plants are imaged at 14 and 21 dpi. (b) Rosette fresh weight analysis of plants inoculated with TuMV‐GFP UK1 at 21 dpi. The aerial part was weighed for five wild‐type mock‐inoculated plants and 15 TuMV‐GFP UK1‐inoculated plants of each genotype. (c) Accumulation analysis of TuMV‐GFP UK1 at 14 dpi. Viral accumulation was detected by double antibody sandwich (DAS)‐ELISA for TuMV coat protein (CP) on six wild‐type mock‐inoculated and at least 16 TuMV‐GFP UK1‐inoculated plants of each genotype. (d) Accumulation analysis of TuMV‐GFP UK1 at 21 dpi. Viral accumulation was detected by DAS‐ELISA for TuMV CP on five wild‐type mock‐inoculated and at least 13 TuMV‐GFP UK1‐inoculated plants of each genotype. Different letters depict significantly different groups identified by Kruskal–Wallis statistical tests at *p* < .05

To assess the resistance generated by disruption of genes encoding translation initiation factors 4E, we compared the phenotypic responses on infection with TuMV‐GFP UK1 of three *Arabidopsis* genotypes: a wild‐type susceptible accession Columbia‐0, a line knocked out for *eIF4E1* (*eif4e1^KO^*), resistant to ClYVV, and a line knocked out for *eIFiso4E* (*eifiso4e^KO^*), resistant to TuMV. The virus‐induced symptoms were recorded at 14 and 21 days postinoculation (dpi) (Figure 1a). Infection of wild‐type plants resulted in typical TuMV symptoms including leaf distortion and stunted growth. On the contrary, *eifiso4e^KO^* plants did not exhibit any disease symptoms at both postinoculation stages, consistent with their previously described full resistance towards TuMV (Duprat et al., 2002). Interestingly, TuMV‐induced disease symptoms in the *eif4e1^KO^* line were clearly more pronounced in comparison to wild‐type plants. Two main phenotypic patterns of this enhanced susceptibility were visible at the leaves formed prior to and after the viral inoculation step in the *eif4e1^KO^* mutant (Figure 1a, leaves formed prior to inoculation are labelled by white arrowheads while leaves formed after the inoculation are labelled by black arrowheads).

The leaves already formed before the inoculation step did not show any visible size or morphology alterations between wild‐type and *eif4e1^KO^* plants. However, as early as 14 dpi, a precocious yellowing was observed in *eif4e1^KO^* plants (Figure 1a, white arrowheads). Imaging total chlorophyll fluorescence suggested a reduction of chlorophyll levels, a trait characteristic of leaf senescence and typically observed at later developmental stages (Pružinská et al., 2005). The tissue senescence in *eif4e1^KO^* plants was even more striking at 21 dpi. While the old leaves of wild‐type plants retained weak chlorophyll fluorescence, tissue chlorosis showed an extensive progression in *eif4e1^KO^* infected plants (Figure 1a, white arrowheads).

The leaf primordia formed after the inoculation step exhibited important malformations such as edge warping and stunted growth in wild‐type plants. The development of these organs seemed to be completely arrested in the *eif4e1^KO^* mutant at both 14 and 21 dpi, resulting in a dwarf‐like rosette morphology (Figure 1a, black arrowheads). Globally, this growth arrest led to an approximately 40% reduction of the weight in *eif4e1^KO^* infected plants (mean rosette fresh weight = 0.22) relative to wild‐type infected plants (mean rosette fresh weight = 0.37; Figure 1b).

Altogether, we conclude that the *Arabidopsis* *eif4e1^KO^* mutant displays strong disease symptoms and increased developmental malformations on TuMV infection.

### 2.2 TuMV accumulation is favoured in the *eif4e1^KO^* mutant

The increased severity of the symptoms induced by TuMV in *eif4e1^KO^* plants prompted us to explore whether this phenotype could be associated with a higher viral load. To compare the accumulation of TuMV, we took advantage of the GFP coding sequence inserted in the TuMV UK1 clone (Beauchemin et al., 2005). Because the signal of GFP fused to TuMV can be used as a proxy for viral accumulation (Abdelkefi et al., 2018), fluorometric analysis with a GFP‐camera was applied as a nondestructive assessment of the viral infection at 14 and 21 dpi.

As expected by the absence of virus disease symptoms, no GFP fluorescence signal was detected in TuMV‐GFP UK1‐inoculated *eifiso4e^KO^* plants. Interestingly, fluorometric analysis of the *eif4e1^KO^* mutant revealed a more intense GFP signal relative to wild‐type plants at 14 and 21 dpi, suggesting an increased viral accumulation in the absence of eIF4E1 (Figure 1a). To validate these differences in TuMV proliferation, we performed a double antibody sandwich (DAS)‐ELISA for TuMV coat protein (CP) detection at the respective postinoculation stages. DAS‐ELISA values for TuMV CP detection were higher at 14 dpi in the *eif4e1^KO^* mutant compared with wild‐type infected plants, although the difference was not statistically supported with *p* < .05 (Figure 1c). This difference was higher and significant for *p* < .05 at 21 dpi corresponding to an approximately 70% increase of the TuMV load in the *eif4e1^KO^* mutant (mean A_405 nm_ = 3.3) compared to wild‐type plants (mean A_405 nm_ = 1.9) (Figure 1d). Hence, GFP imaging and DAS‐ELISA assays both suggest that *eif4e1^KO^* mutant plants infected with the TuMV‐GFP UK1 isolate accumulate more viral proteins.

**FIGURE 2:**
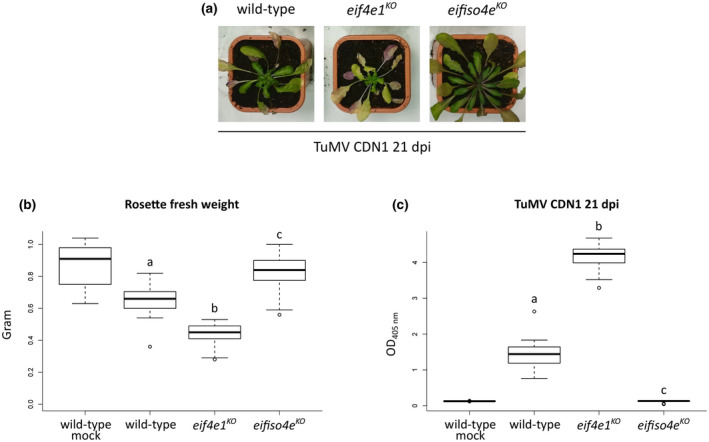
The enhanced susceptibility of the *Arabidopsis* *eif4e1^KO^*mutant extends to the CDN1 isolate of turnip mosaic virus (TuMV). (a) Phenotypic comparison of representative plants on TuMV CDN1 infection 21 days postinoculation (dpi). (b) Rosette fresh weight analysis of plants inoculated with TuMV CDN1 at 21 dpi. The aerial part was weighed for five wild‐type mock‐inoculated plants and at least 13 TuMV CDN1‐inoculated plants of each genotype. (c) Accumulation analysis of TuMV CDN1 at 21 dpi. Viral accumulation was detected by double antibody sandwich‐ELISA for TuMV coat protein (CP) on five wild‐type mock‐inoculated and at least 13 TuMV CDN1‐inoculated plants of each genotype. Different letters depict significantly different groups identified by Kruskal–Wallis statistical tests at *p* < .05

Phenotypic response to TuMV infection is known to be isolate‐dependent as different TuMV isolates induce contrasting disease symptoms (Sanchez et al., 2015). To test if the developmental defects and the higher virus load observed in the *eif4e1^KO^* mutant occur specifically in response to infection with the TuMV‐GFP UK1 isolate, we performed an inoculation test with the CDN1 isolate of TuMV (Duprat et al., 2002). Once again, the *eif4e1^KO^* mutant was more strongly affected than wild‐type plants (Figure 2a). As observed with TuMV‐GFP UK1 inoculation, *eif4e1^KO^* plants infected by TuMV CDN1 displayed an approximately 33% decrease in weight (mean fresh rosette weight = 0.43) compared to wild‐type plants (mean fresh rosette weight = 0.65; Figure 2b). Importantly, the accumulation of TuMV CDN1 was about three times higher in *eif4e1^KO^* than in wild‐type inoculated plants (Figure 2c). Therefore, the enhancement of viral accumulation extends to the TuMV CDN1 isolate, suggesting that the over‐accumulation of TuMV in *eif4e1^KO^* is consistent and not isolate‐dependent, although it would be of interest to test more TuMV isolates.

Taken together, these results suggest that the accumulation of TuMV is favoured in the absence of *eIF4E1*.

### 2.3 Complementation of *eIF4E1* loss of function restores wild‐type susceptibility to TuMV

**FIGURE 3:**
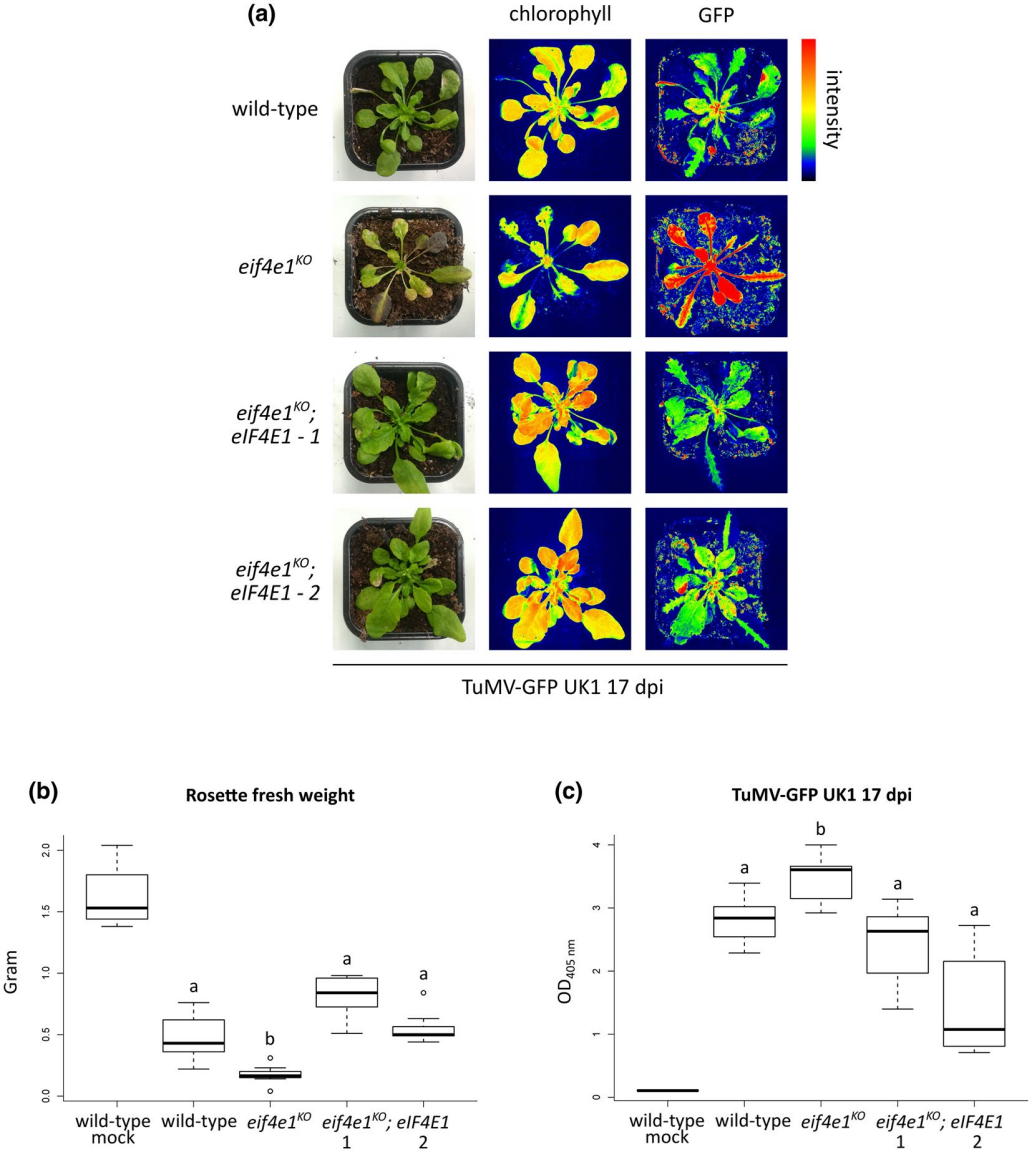
Complementation of *eIF4E1* loss of function suppresses the enhanced susceptibility towards turnip mosaic virus (TuMV). (a) Phenotypic comparison of representative plants upon TuMV‐GFP UK1 infection at 17 days postinoculation (dpi). Photographs were taken under natural light conditions (left panel) and under wavelengths specific for chlorophyll excitation (middle panel) or green fluorescent protein (GFP) excitation (right panel) by using GFP Camera (PSI) fluorescence imaging. Fluorescence is represented by false colours ranging from blue (low intensity) to red (high intensity). Two independently obtained *eif4e1^KO^;eIF4E1* complemented lines were used in the analyses. (b) Rosette fresh weight analysis of plants inoculated with TuMV‐GFP UK1 at 17 dpi. The aerial part was weighed for five wild‐type mock‐inoculated plants and at least seven TuMV‐GFP UK1‐inoculated plants of each genotype. (c) Accumulation analysis of TuMV‐GFP UK1 at 17 dpi. Viral accumulation was detected by double antibody sandwich‐ELISA for TuMV coat protein (CP) on five wild‐type mock‐inoculated and at least seven TuMV‐GFP UK1‐inoculated plants of each genotype. Different letters depict significantly different groups identified by Kruskal–Wallis statistical tests at *p* < .05

To confirm that the *eif4e1^KO^* severe developmental phenotype is caused by the lack of eIF4E1, we checked the effect of TuMV infection on *eif4e1^KO^* plants complemented by a full‐length genomic *eIF4E1* under the control of its native promoter and 3′ untranslated region (UTR) (Bastet et al., 2018). Two independently obtained *eIF4E1*‐complemented lines, hereafter referred to as *eif4e1^KO^; eIF4E1‐1* and *eif4e1^KO^; eIF4E1*‐*2*, were inoculated with TuMV‐GFP UK1 and their disease symptoms were analysed at 17 dpi, a medium time point between 14 and 21 dpi. The complementation of the *eIF4E1* loss of function resulted in a wild‐type susceptibility phenotype on infection: the phenotypic patterns regarding leaves formed prior to and after the inoculation step resembled those of wild‐type inoculated plants and not of *eif4e1^KO^* inoculated plants (Figure 3a). Consistently, the rosette fresh weights of both *eif4e1^KO^*; *eIF4E1‐1* and *eif4e1^KO^*; *eIF4E1*‐*2* complemented lines were similar to wild‐type, while *eif4e1^KO^* displayed about 64% weight reduction (mean fresh rosette weight = 0.17) compared with wild‐type plants (mean fresh rosette weight = 0.48) on TuMV‐GFP UK1 infection (Figure 3b). Finally, while TuMV accumulation was enhanced in the *eif4e1^KO^* mutant, the complemented *eif4e1^KO^*; *eIF4E1‐1* and *eif4e1^KO^*; *eIF4E1*‐*2* lines exhibited viral protein levels similar to wild‐type plants (Figure 3c). Similar results were obtained when the analyses were repeated at 14 and 21 dpi (Figures S1 and S2).

Hence, the complementation of *eif4e1^KO^* function is sufficient to revert to a wild‐type susceptibility to TuMV, showing that disruption of *eIF4E1* is the sole cause for the increased susceptibility and higher virus load in the *eif4e1^KO^* mutant.

### 2.4 TuMV overaccumulation is not associated with a change in eIFiso4E protein accumulation in the *eif4e1^KO^* mutant

Previously, it was demonstrated that an *eIFiso4E* knockout allele causes an enhanced accumulation of eIF4E1 protein in *Arabidopsis*, suggesting the existence of a regulatory mechanism among eIF4E family members (Duprat et al., 2002). We hypothesized that the difference in viral accumulation in *eif4e1^KO^* could be due to a difference in the accumulation of eIF4E factors. Hence, a greater amount of eIFiso4E in *eif4e1^KO^* lines could provide a more abundant host factor for TuMV and possibly favour viral accumulation.

**FIGURE 4:**
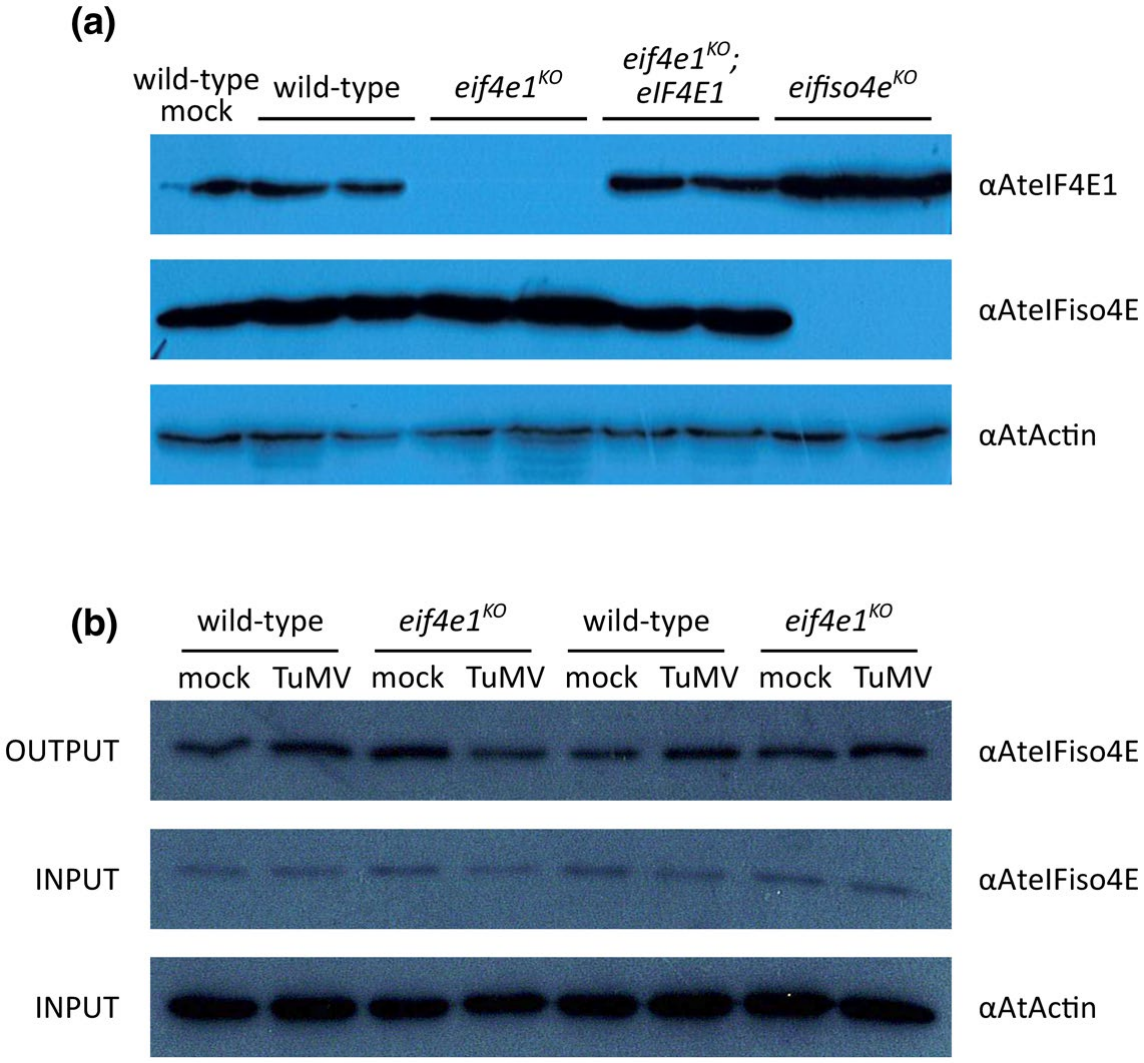
The eIFiso4E protein levels are not increased in the *eif4e1^KO^* mutant. (a) Western blot analysis on total protein extracts from mock‐inoculated wild‐type and TuMV‐GFP UK1‐inoculated plants at 21 days postinoculation (dpi). eIF4E1 and eIFiso4E protein accumulation was analysed by western blot on total plant protein extracts using specific antibodies. Equal loading was checked by western blot using anti‐actin antibodies. (b) Western blot analysis on m^7^GTP‐purified protein extracts from mock‐ or TuMV‐GFP UK1‐inoculated wild‐type and *eif4e1^KO^* plants at 21 dpi. Total protein extracts (INPUT) were pulled down with γ‐aminophenyl‐m^7^GTP (C_10_‐spacer)‐agarose beads (OUTPUT). After purification, the output fraction was analysed by western blot using anti‐eIFiso4E antibody while equal loading control was checked on total protein extracts (INPUT) by western blot using anti‐actin antibodies. Each lane represents immunoblotted protein extracts obtained from a single independent plant

To address this question, we compared the accumulation of eIF4E1 and eIFiso4E in the different *Arabidopsis* genotypes in the presence of TuMV infection. Western blot analysis revealed higher eIF4E1 protein levels in *eifiso4e^KO^* (Figure 4a) corresponding to an approximately 50% increase as determined by band intensity quantification (see Figure S3a,b for short‐exposure images and band intensity quantification), confirming the results demonstrated by previous studies (Duprat et al., 2002). However, no changes in eIFiso4E accumulation could be detected between wild‐type and *eif4e1^KO^* (Figures 4a and S3c).

To examine whether a subtler variation in eIFiso4E levels occurs in *eif4e1^KO^*, we performed an m^7^GTP pull‐down on protein extracts obtained from wild‐type and *eif4e1^KO^* mutant plants. This allows selective purification of the proteins associated with the cap‐binding complexes in the total plant extract, including eIF4E and eIFiso4E. Western blot analysis on m^7^GTP‐bound fractions (OUTPUT) confirmed the presence of eIFiso4E proteins but no change in its accumulation pattern was observed in the *eif4e1^KO^* mutant relative to wild‐type plants in the absence or presence of TuMV infection (Figure 4b).

These results rule out that a significant increase in eIFiso4E protein level could directly explain the overaccumulation of TuMV in *eif4e1^KO^*.

### 2.5 Virus evolution is not responsible for the enhanced susceptibility of the *eif4e1^KO^* mutant towards TuMV

The viral genome‐linked protein (VPg) of potyviruses has been identified as a key virulence determinant of potyvirus infection. The amino acid substitutions E116Q and N163Y in the central and C‐terminal part of TuMV VPg are known to expand the target range of TuMV in *Arabidopsis* by allowing it to recruit the eIF4E1 factor and consequently to overcome resistance mediated by *eIFiso4E* loss of function (Bastet et al., 2018; Gallois et al., 2010). In addition to this qualitative effect on virus pathogenicity, nonsynonymous changes in the VPg of tobacco etch virus (TEV) lead to an increase in virus aggressiveness (i.e., the severity of disease symptoms) and favoured virus accumulation (Agudelo‐Romero et al., 2008). Based on these observations, one could speculate that multiplication of TuMV in the absence of *eIF4E1* could result in a similar gain of aggressiveness by the acquisition and selection of specific mutations at the VPg.

**FIGURE 5:**
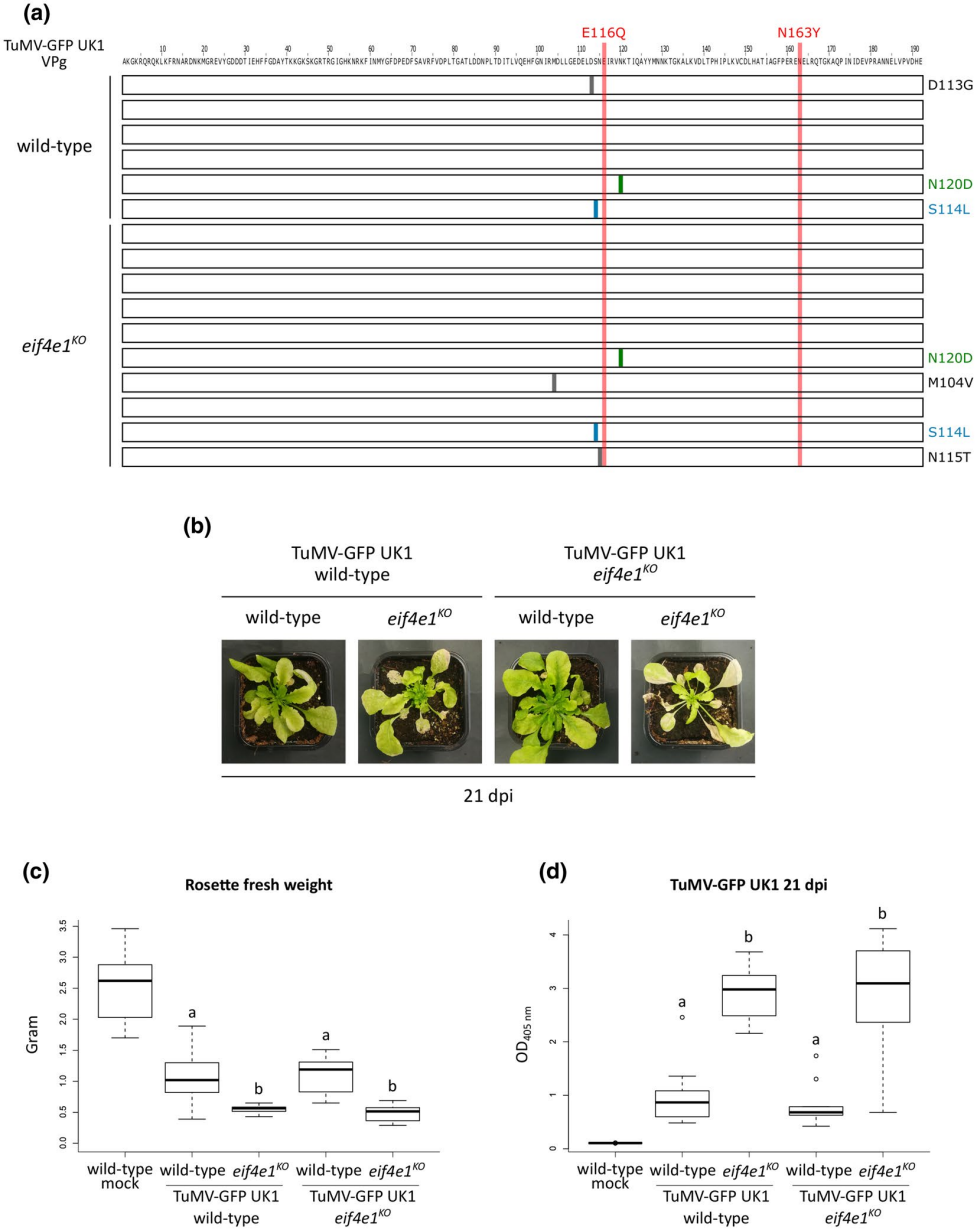
The increased susceptibility of the *eif4e1^KO^* mutant towards turnip mosaic virus (TuMV) is not caused by virus evolution. (a) A graphic representation of nonfixed amino acid substitutions at TuMV VPg following TuMV‐GFP UK1 multiplication on wild‐type and *eif4e1^KO^* plants. Total RNA extracted from six wild‐type and 10 *eif4e1^KO^* TuMV‐GFP UK1‐inoculated plants was reverse transcribed and the VPg coding region was sequenced. Each box represents a VPg sequence obtained from an independent wild‐type or *eif4e1^KO^* TuMV‐GFP UK1‐inoculated plant. Vertical lines indicate the positions of the detected nonfixed amino acid substitutions. The VPg polymorphisms N120D and S114L observed in both wild‐type and *eif4e1^KO^* are represented in green and blue, respectively. The positions of amino acid substitutions associated with *eifiso4e^KO^* resistance breaking (Gallois et al., 2010) are highlighted in red. (b) Phenotypic comparison of representative plants upon back‐inoculation with TuMV‐GFP UK1 multiplied on either wild‐type or *eif4e1^KO^* 21 days postinoculation (dpi). (c) Rosette fresh weight analysis of plants back‐inoculated with TuMV‐GFP UK1 multiplied on either wild‐type or *eif4e1^KO^* 21 dpi. The aerial part was weighed for nine wild‐type mock‐inoculated plants and at least nine TuMV‐GFP UK1‐back inoculated plants of each genotype. (d) Accumulation analysis of back‐inoculated TuMV‐GFP UK1 at 21 dpi. Viral accumulation was detected by double antibody sandwich‐ELISA for TuMV coat protein (CP) on at least nine TuMV‐GFP UK1‐back inoculated plants of each genotype. Different letters depict significantly different groups identified by Kruskal–Wallis statistical tests at *p* < .05

To assess whether multiplication of TuMV in *eif4e1^KO^* favours the acquisition of specific mutations in the VPg, we compared the sequences of TuMV VPg encoded by viral RNA extracted from wild‐type and *eif4e1^KO^* TuMV‐GFP UK1‐inoculated plants. First, this analysis revealed that most of the sequences (i.e., three out of the six obtained from wild‐type inoculated plants and six out of the 10 obtained from *eif4e1^KO^* inoculated plants) did not display any polymorphism in the VPg coding sequence (Figure 5). Second, none of the mutations detected in the remaining VPg sequences were fixed in the viral populations (Table S1). Furthermore, identical VPg polymorphisms resulting in the nonsynonymous amino acid substitutions N120D and S114L were present in the viral progeny obtained from both wild‐type or *eif4e1^KO^* inoculated plants, suggesting no preferential selection caused by the plant genotype (Figure 5a, green and blue vertical lines). Importantly, none of the nucleotide changes in the VPg sequenced from wild‐type and *eif4e1^KO^* infected plants corresponded to the E116Q and N163Y substitutions associated with resistance breaking (Bastet et al., 2018; Gallois et al., 2010). Altogether, these results suggest that TuMV multiplied in the *eif4e1^KO^* mutant did not acquire distinct VPg mutations compared to TuMV multiplied in wild‐type plants.

To determine whether propagation of TuMV in *eif4e1^KO^* results in an increase of aggressiveness in a VPg‐independent manner, extracts from either wild‐type or *eif4e1^KO^* infected plants were back‐inoculated to the same two genotypes and plant susceptibility was scored at 21 dpi. Our results show that whether the TuMV back‐inoculum originated from wild‐type or from *eif4e1^KO^* plants, severe disease symptoms were observed only in *eif4e1^KO^* plants (Figure 5b). Accordingly, the strong weight reduction and increased viral loads, characteristic of the TuMV hypersusceptibility, were found only in *eif4e1^KO^* back‐inoculated plants (Figure 5b,c). Therefore, the susceptibility outcome towards TuMV depends on the genotype of the back‐inoculated plants, regardless of the origin of the inoculum.

These results demonstrate that no selection pressure is imposed by the absence of *eIF4E1*, ruling out the hypothesis that the hypersusceptibility of *eif4e1^KO^* is caused by direct evolution of the virus affecting its aggressiveness. Moreover, back‐inoculation further confirms the high TuMV accumulation and increased disease symptoms associated with *eIF4E1* loss of function.

### 2.6 An edited *eIF4E1^N176K^* resistance allele, encoding a functional protein, does not trigger an increased susceptibility towards TuMV

While generating KO in *eIF4E1* is efficient to drive resistance to ClYVV (Bastet et al., 2018; Sato et al., 2005) that relies on this translation initiation factor in *Arabidopsis*, we show here that such an approach comes at the price of an increased susceptibility to a virus targeting the *eIFiso4E* paralog. As previously suggested (Bastet et al., 2017), we reasoned that an alternative option would be to mimic functional resistance *eIF4E1* alleles rather than knocking them out. In our previous work, we used the CRISPR‐nCas9‐cytidine deaminase method to convert the *Arabidopsis* *eIF4E1* susceptibility allele to ClYVV into a functional resistance allele in a transgene‐free manner (Bastet et al., 2019). The protein encoded by this allele contains the single amino acid substitution N176K, imitating a mutation that occurs naturally in a pea *eIF4E* allele associated with resistance to potyviruses. When modified in the wild‐type *Arabidopsis* *eIF4E1* gene, it confers complete resistance to two distinct ClYVV isolates while not impairing the plant's development. Here, we investigated whether this resistance allele, hereafter referred to as *eIF4E1^N176K^,* is able to circumvent the increased disease symptoms associated with the knockout of *eIF4E1* in response to TuMV infection.

**FIGURE 6:**
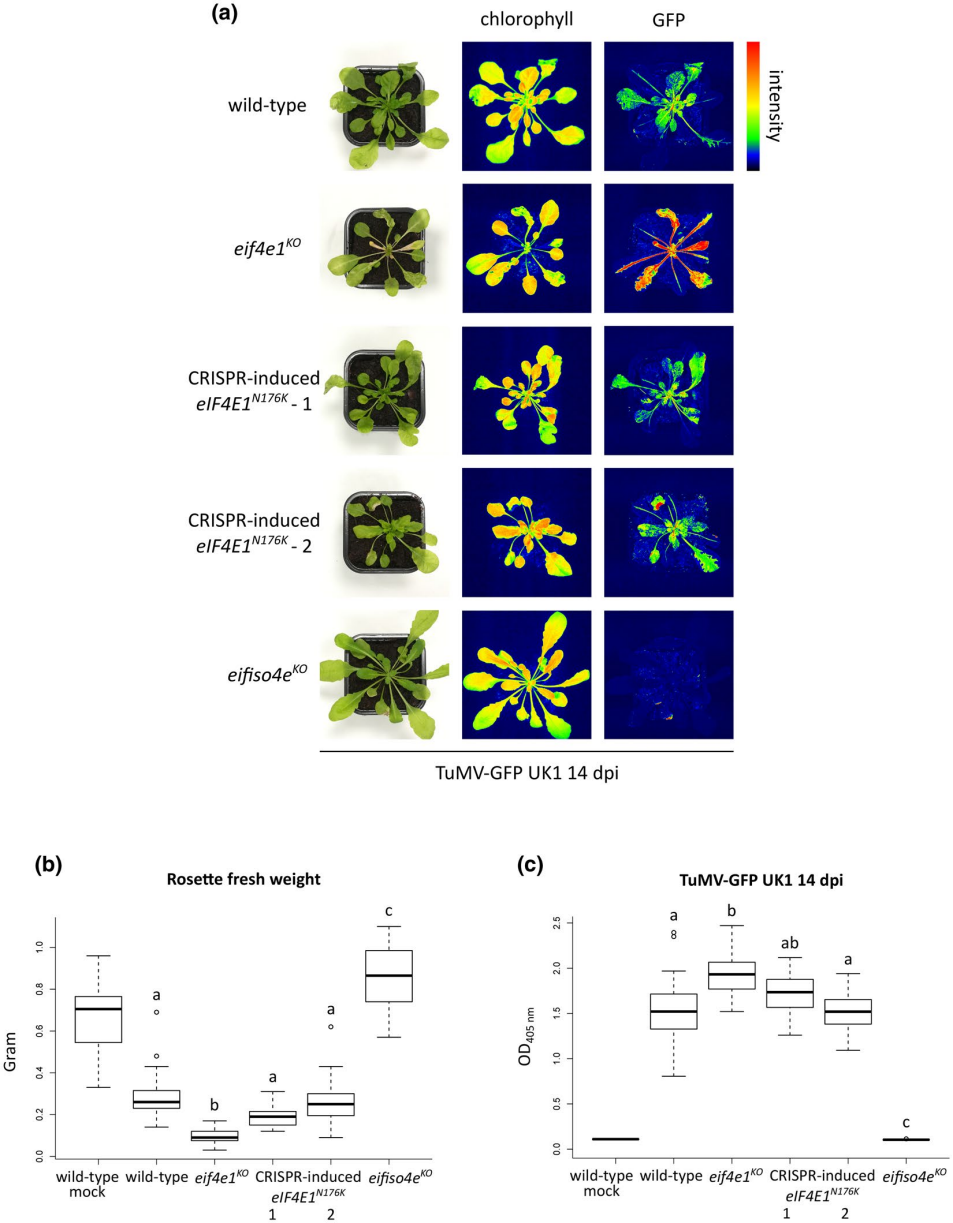
The functional *eIF4E1^N176K^* resistance allele does not trigger hypersusceptibility to turnip mosaic virus (TuMV). (a) Phenotypic comparison of representative plants upon TuMV‐GFP UK1 infection at 14 days postinoculation (dpi). Photographs were taken under natural light conditions (left panel) and under wavelengths specific for chlorophyll excitation (middle panel) or green fluorescent protein (GFP) excitation (right panel) by using GFP Camera (PSI) fluorescence imaging. Fluorescence is represented by false colours ranging from blue (low intensity) to red (high intensity). Two independently obtained CRISPR‐induced *eIF4E1^N176K^* lines were used in the analyses. (b) Rosette fresh weight analysis of plants inoculated with TuMV‐GFP UK1 at 18 dpi. The aerial part was weighed for 24 wild‐type mock‐inoculated plants and at least 23 TuMV‐GFP UK1‐inoculated plants of each genotype. (c) Accumulation analysis of TuMV‐GFP UK1 at 14 dpi. Viral accumulation was detected by double antibody sandwich‐ELISA for TuMV coat protein (CP) on 24 mock‐inoculated and 24 TuMV‐GFP UK1‐inoculated plants of each genotype. Different letters depict significantly different groups identified by Kruskal–Wallis statistical tests at *p* < .05

To answer this question, we examined the susceptibility outcome of two independently obtained CRISPR‐induced *eIF4E1^N176K^* lines upon TuMV infection. Fourteen days postinoculation the phenotype of the *eIF4E1^N176K^*‐expressing lines was indistinguishable from wild‐type plants (Figure 6a). The accelerated tissue senescence at older leaves, characteristic of *eif4e1^KO^* on TuMV infection, was not observed and the development of newly formed primordia was similar to wild‐type inoculated plants. While *eif4e1^KO^* plants displayed a significant weight loss on TuMV infection, the reduction of weight in *eIF4E1^N176K^*‐expressing plants was similar in comparison to wild‐type plants (Figure 6b). Following inoculation with TuMV‐GFP UK1, plants harbouring the *eIF4E1^N176K^* allele showed a level of GFP intensity similar to wild‐type rather than the enhanced signal imaged in *eif4e1^KO^* plants (Figure 6a). In accordance, the accumulation of TuMV CP was lower in the *eIF4E1^N176K^* lines than in the *eif4e1^KO^* plants (Figure 6c). This suggests that the *eIF4E1^N176K^* resistance allele does not trigger TuMV overaccumulation as the knockout of *eIF4E1* does. Similar results were observed at a later stage of infection such as 21 dpi (Figure S4).

These results demonstrate that a functional resistance allele tailored with CRISPR/Cas9 base‐editing technology is able to avoid the increased susceptibility to TuMV imposed by the knockout in *eIF4E1*, while providing resistance to ClYVV. Hence, it appears that tailored functional *eIF4E* alleles allow the generation of resistance while circumventing drawbacks associated with knocking out these genes.

## 3 DISCUSSION

Knocking out genes encoding factors required for the infection cycle of potyviruses appears to be a straightforward approach to achieve resistance (Mäkinen, 2020; Pyott et al., 2020). Here, our results demonstrate that such a strategy can be double edged as besides providing resistance to ClYVV, knocking out *eIF4E1* makes plants especially vulnerable to infection by TuMV. As it stands, this is the first time such an Achilles’ heel has been experimentally shown for eIF4E‐based resistance to viruses in plants. Our report reinforces the idea that resistance based on disrupting *eIF4E* alleles should be avoided (Bastet et al., 2017) and could have important mechanistic and practical implications in plant breeding.

What causes the enhanced susceptibility of the *Arabidopsis* *eif4e1^KO^* mutant towards TuMV is an intriguing question. The initiation of translation in plants is a tightly regulated process dependent on two distinct cap‐binding complexes: the eIF4F complex (composed of the cap‐binding subunit eIF4E and the scaffolding subunit eIF4G) and the eIFiso4F complex (composed of the eIFiso4E and eIFiso4G counterparts) (Browning & Bailey‐Serres, 2015). The disruption of a single gene coding for the cap‐binding 4E or the scaffolding 4G subunits is known to impact the accumulation of their counterparts, suggesting the existence of a feedback mechanism that regulates the homeostasis of translation initiation factors in the plant cell (Combe et al., 2005; Duprat et al., 2002; Lellis et al., 2010). In the light of these observations, a disruption of eIF4E homeostasis due to the absence of eIF4E1 could lead to a compensatory increase of eIFiso4E accumulation. Higher eIFiso4E levels could supply a more abundant host factor for TuMV accumulation, and therefore explain the enhanced susceptibility of the *eif4e1^KO^* mutant. We ruled out this hypothesis by demonstrating that there was no significant difference of eIFiso4E protein accumulation between *eif4e1^KO^* and wild‐type plants (Figures 4 and S3).

However, even if the absence of *eIF4E1* does not directly affect the overall accumulation of eIFiso4E, it could still be responsible for a more available eIFiso4E target for TuMV accumulation. It is noteworthy that both the eIF4E cap‐binding and the eIF4G scaffolding components of the cap‐binding complexes are required for the establishment of potyvirus infection, suggesting that the entire eIF4F/eIFiso4F complex is recruited during the potyvirus infection cycle (Nicaise et al., 2007). Several studies have put forward the idea that rearrangements in the composition of these complexes could modulate the availability of eIF4E factors for potyviruses. Kang et al. (2007) first proposed that the ectopic expression of the pepper *eIF4E* resistance allele *pvr1* in a susceptible tomato accession could confer potyvirus resistance by saturating the cellular pool of eIF4G proteins with an eIF4E factor that cannot be recruited by the virus, and thus render endogenous eIF4E susceptibility factors inaccessible for viral multiplication. In a similar way, Gauffier et al. (2016) showed that in the wild tomato species *Solanum habrochaites*, the *Sh‐eIF4E1^PI24^‐pot1* resistance allele acts dominantly over the *eIF4E2* susceptibility allele. The molecular basis of the dominant effect of *Sh‐eIF4E1^PI24^‐pot1* over *eIF4E2* is not yet understood but could be explained by an enrichment of the cellular pool of eIF4F complexes with the Sh‐eIF4E1^PI24^‐pot1 protein inadequate for viral multiplication. It is known that mixed cap‐binding complexes between eIFiso4E and eIF4G occur in wheat germ extracts and are fully capable of supporting the translation of alfalfa mosaic virus (AMV) RNA (Mayberry et al., 2011). Hence, one could imagine a scenario where the loss of *eIF4E1* reshuffles the distribution of eIFiso4E in the translational initiation machinery by the formation of mixed complexes between eIFiso4E and eIF4G. Such mixed complexes could provide a more available eIFiso4E target for TuMV accumulation and explain the increase of susceptibility in the absence of eIF4E1. Exploring the mechanism behind the hypersusceptibility of the *eif4e1^KO^* mutant towards TuMV could help to understand better the homeostasis between eIF4E factors in the plant cell and what makes them available to potyviruses.

Our results reveal that besides supplying resistance to viruses, the withdrawal of eIF4E susceptibility factors comes at the expense of an unanticipated increase in susceptibility. Interestingly, a similar trade‐off between broad resistance spectrum and resistance efficiency occurs in pepper. In the ‘Perennial’ cultivar of pepper, a deletion of 82 nucleotides in the *pvr6* locus leading to a disruption of an *eIFiso4E* gene has been shown to provide resistance to chilli veinal mottle virus (ChiVMV) and pepper veinal mottle virus (PVMV) when combined with *pvr2* alleles encoding functional eIF4E factors (Caranta, 1997; Moury et al., 2005; Rubio et al., 2008). An unexpected drawback of this gain of resistance was illustrated by Quenouille et al. (2014) who demonstrated that the natural knockout allele *pvr6* was also linked to an increased resistance breaking of the *pvr2^3^* resistance allele by PVY. Moreover, it was found that this higher resistance breakdown frequency correlated strongly with higher PVY loads in *pvr6*‐carrying pepper accessions (Quenouille et al., 2016). Although the direct involvement of *eIFiso4E* in these events remains to be functionally validated, it appears that in the pepper–PVY pathosystem, *eIFiso4E* absence could favour viral accumulation in a way reminiscent of how *eIF4E1* loss of function triggers TuMV overaccumulation in the current model. These results mirror our findings by suggesting that although supplying resistance, using knockout alleles in breeding programmes could decrease the resistance efficiency towards viruses for which multiplication does not rely on the mutated factor.

Another important setback of resistance mediated by knocking out *eIF4E* has been exemplified in tomato in which the potyviruses PVY and TEV require the *eIF4E1* and *eIF4E2* factors for multiplication. While a TILLING‐induced disruption in *eIF4E1* generated resistance to a single PVY isolate, a naturally occurring functional eIF4E1 counterpart *Sh‐eIF4E1^PI24^‐pot1* enlarged the resistance spectrum to several isolates of PVY and TEV (Gauffier et al., 2016). Indeed, a wide resistance spectrum to these isolates could be achieved by disrupting both *eIF4E1* and *eIF4E2* factors but had a severe effect on plant development, whereas the functional *eIF4E1^PI24^* allele did not impose any fitness cost. Similarly, we show that the increased susceptibility towards TuMV governed by *eIF4E1* knockout can be avoided by using a functional *eIF4E1^N176K^* allele that retains a wide spectrum resistance to several potyviruses (Bastet et al., 2019) (Figure 6). These direct comparisons of two strategies illustrate the superiority of functional resistance alleles over knockout *eIF4E* alleles in the generation of efficient resistance to potyviruses.

In addition to suggesting arguments for the long‐term inefficiency of resistance mediated by knocking out *eIF4E* factors, the cases in tomato and pepper described above raise the question of how regulation between eIF4E could play a role in resistance to potyviruses. Knocking out eIF4E family members appears to disrupt the tight homeostasis between these proteins and to have direct repercussions on their availability for viruses (Michel et al., 2019). Indeed, the large majority of resistance alleles identified by studying natural variation encode functional proteins and only a very few alleles coding for nonfunctional proteins have been identified, and only for eIFiso4E (for review, see Bastet et al., 2017; Wang & Krishnaswamy, 2012). It has been suggested that natural *eIF4E* loss of function alleles are not selected as their inactivation may be associated with adverse developmental defects but also because they could be of limited use for providing resistance to viruses: this is illustrated by the analysis of engineered *eIF4E* KO alleles especially in tomato and *Arabidopsis* (Bastet et al., 2017; Gallois et al., 2010; Gauffier et al., 2016). The advent of CRISPR‐based gene inactivation will allow such studies to be extended in new species. It will be of major interest to assess the long‐term effects on the plant development, resistance spectrum, and resistance durability for recently developed resistances based on *eIF4E* knockout in cucumber (*Cucumis sativa*) and cassava, for example (Chandrasekaran et al., 2016; Gomez et al., 2019). An alternative to developing durable and broad‐spectrum resistances to potyviruses may, however, reside in the use of naturally occurring functional *eIF4E* resistance alleles. Novel gene‐editing technologies such as CRISPR/Cas9 coupled with Cas9 cytidine and adenine deaminase fusions represent a promising approach for the engineering of resistance by the precise editing of susceptibility alleles in crops in which natural variability is lacking (Mushtaq et al., 2019; Veillet et al., 2020).

In conclusion, our findings reinforce the idea, initially built on observations made in the major crops tomato and pepper, that resistance based on knocking out *eIF4E* factors should be avoided in plant breeding. Here, results gathered in *Arabidopsis* suggest that such a strategy could expose the plant to the severe threat of potyviruses able to recruit alternative *eIF4E* copies. At the same time, it provides a simple model to explore the mechanism behind the availability of eIF4E factors to viruses; understanding this could help to make resistance more efficient in crops. It is important to question whether this observation is restricted to the small multigene families of translation initiation factors or if it can be generalized to other susceptibility factors.

## 4 EXPERIMENTAL PROCEDURES

### 4.1 Plant and virus material

*A. thaliana* ecotype Columbia‐0 (Col‐0) was used as the wild‐type accession for all experiments. Mutations in the *eif4e1^KO^* and *eifiso4e^KO^* homozygous lines were caused by a T‐DNA insertion in *eIF4E1* (At1g18040; SALK_145583) and a dSpm transposon insertion in *eIFiso4E* (At5g35620; Duprat et al., 2002), respectively. Both mutations are in the Columbia‐0 (Col‐0) background. The complemented *eif4e1^KO^; eIF4E1* lines carries a genomic At4g18040 *eIF4E1* fragment (spanning 1,500 bp of the promoter region and 150 bp of the 3′ UTR) (Bastet et al., 2018). Two independently obtained T_3_ *eif4e1^KO^;eIF4E1* lines were used in the analyses. The CRISPR‐nCas9‐cytidine deaminase‐induced *eIF4E1^N176K^* lines were obtained by agrotransformation of Col‐0 plants with the pDICAID_nCas9‐PmCDA_NptII_eIF4E1 construct as previously reported (Bastet et al., 2019). Two independently obtained transgene‐free T_4_ CRISPR‐induced *eIF4E1^N176K^* lines were used in the analyses. The TuMV CDN1 isolate was used as previously reported (Duprat et al., 2002). For TuMV‐GFP UK1 infection, a binary vector was kindly provided by Jean‐François Laliberté (Beauchemin et al., 2005).

### 4.2 Plant culture and virus inoculation

Plant seeds were directly sowed on soil or on Murashige and Skoog (MS) medium following surface sterilization for 10 min in 95% ethanol and 0.1% Tween 20. The complemented *eif4e1^KO^;eIF4E1* plants were selected for transgene presence on MS plates supplemented with 5 mg/L hygromycin B. Two weeks after sowing, seedlings were individually transferred to soil and cultivated in a culture chamber at 20–24 °C with a short‐day cycle of 8 hr light (100 µmol⋅m^−2^ s^−1^) and 16 hr dark.

Prior to plant inoculation, the TuMV CDN1 and TuMV‐GFP UK 1 isolates were propagated on turnip plants (*Brassica rapa*). Propagation of the TuMV CDN1 isolate was performed by rub‐inoculating turnip plants with dried TuMV CDN1‐infected turnip leaves ground in phosphate buffer (0.03 M Na_2_HPO_4_, 0.2% diethyldithiocarbamate [DIECA], pH 7) containing carborundum and active carbon. Propagation of the TuMV‐GFP UK1 isolate was performed by rub‐inoculating turnip plants with a suspension of the *Agrobacterium tumefaciens* C58S pMP90 carrying pCambia TuMV‐GFP (Beauchemin et al., 2005). The *Agrobacterium* suspension was prepared by growing the bacteria in Luria–Bertani liquid medium supplemented with 50 mg/ml kanamycin, 25 mg/ml gentamicin, and 50 mg/ml rifampicin for 48 hr, and then suspending in 10 mM MgCl_2_/MES (2‐[*N*‐morpholino]ethanesulfonic acid) to an optical density of 0.8 units at 600 nm.

One or two young leaves of 4‐week‐old *Arabidopsis* plants were rub‐inoculated by either the TuMV CDN1 or TuMV‐GFP UK1 isolates propagated on turnip plants following grinding of fresh turnip leaves in phosphate buffer containing active carbon and carborundum.

### 4.3 Fluorometric camera analysis

Monitoring of TuMV‐GFP infection was carried out using a closed fluorometric camera FluorCam FC 800‐C/1010‐GFP (Photon System Instruments) equipped with chlorophyll and GFP filters. Leaf senescence was determined by measuring steady‐state chlorophyll fluorescence with constant illumination. GFP fluorescence was captured by the camera on excitation at 395 nm. Images were obtained by using FluorCam7 v. 1.2.5.3 software (Photon System Instruments). Fluorescence is represented in false colours from blue (low intensity) to red (high intensity).

### 4.4 Fresh weight and virus accumulation analyses

The fresh weight of individual plants was determined by cutting and weighing the aerial part of plants at the respective postinoculation stage.

Virus accumulation was assessed by DAS‐ELISA at the respective postinoculation stage. The weighed aerial part of individual plants was ground with an adjusted volume of phosphate buffer (4 ml of phosphate buffer per 1 g of fresh weight). Ground plant extracts were deposited on a 96‐well uncoated ELISA plate and incubated overnight at 4 °C. Plates were washed three times with permutated water and once with phosphate‐buffered saline‐Tween and diluted detection antibody (1:200) directed against the coat protein (CP) of TuMV was added (Agdia). Following 2 hr incubation at 37 °C, the plate was washed as described above and diluted alkaline phosphatase‐conjugated secondary antibody (1:200) was added. Plates were incubated at room temperature for 2 hr and after a wash step, *p*‐nitrophenylphosphate substrate was added. Plate reads were carried out by spectrophotometry at 405 nm periodically in the 30 min following substrate addition.

### 4.5 Total protein extraction and m^7^GTP pull‐down assay

For total protein analysis, extracts were prepared by grinding equal amounts of TuMV‐GFP UK1 systemically infected leaves in Laemmli buffer and boiling samples for 5 min. Proteins contained in the supernatant were recuperated following centrifugation for 10 min at 15,000 × g. For the m^7^GTP pull‐down assay, total protein extracts were prepared by grinding whole mock or TuMV‐GFP UK1‐inoculated plants in liquid nitrogen and suspending 100 mg homogenized tissue in a binding buffer (40 mM HEPES/KOH pH 7.6, 100 mM KCl, 1 mM dithiothreitol, 10% glycerol, 1% phenylmethanesulphonyl fluoride and 1 × protease inhibitor cocktail [Roche]). After centrifugation for 10 min at 15,000 × g at 4 °C, the protein‐containing supernatant fraction (INPUT fraction) was recovered and incubated at 4 °C overnight with 50 µl of prewashed immobilized γ‐aminophenyl‐m^7^GTP (C_10_‐spacer)‐agarose beads (Jena Bioscience). To remove any unbound proteins, the m^7^GTP‐agarose beads were centrifuged at for 1 min at 15,000 × g and washed four times with the binding buffer at 4 °C. Finally, the proteins linked to the m^7^GTP‐agarose beads were eluted by boiling in 50 µl Laemmli buffer (OUTPUT fraction).

### 4.6 Western blotting

Equal amounts of protein extracts were electrophoresed on 12% sodium dodecyl sulphate (SDS) polyacrylamide gel prior to transfer on Hybond ECL nitrocellulose membranes (GE Healthcare). Membranes were blocked overnight in Tris‐buffered saline containing 5% dry milk (TBS + 5% milk) and incubated with rabbit polyclonal antibodies directed against *Arabidopsis* eIF4E1 (diluted 1:2,000 in TBS + 5% milk) (Bastet et al., 2018), eIFiso4E (diluted 1:2,500 in TBS + 5% milk) (Estevan et al., 2014), and a mouse monoclonal antibody directed against plant actin (diluted 1:5,000 in TBS + 5% milk) (Sigma‐Aldrich). Following three washes with TBS supplemented with 0.1% Tween 20, membranes were probed with secondary goat horseradish peroxidase‐linked anti‐rabbit serum for detection of eIF4E1 and eIFiso4E (diluted 1:2,000 in TBS + 5% milk) and a goat horseradish peroxidase‐linked anti‐mouse serum for detection of actin (diluted 1:5,000 in TBS + 5% milk). Detection of horseradish peroxidase activity was performed using a LumiGLO Reserve chemiluminescent substrate kit (SeraCare) and X‐OMAT LS films (Kodak).

### 4.7 VPg cDNA sequence analysis

Total RNA was extracted from TuMV‐GFP UK1 systemically infected leaves using TRI‐reagent (Sigma‐Aldrich). Reverse transcription was performed on 500 ng RNA using a PrimeScript RT reagent kit (Takara) with oligo‐dT and random hexamer primers. The primer pair 5′‐GCGAAAGGTAAGAGGCAAAGG‐3′ (forward) and 5′‐CTCGTGGTCCACTGGGACGA‐3′ (reverse) was used to amplify a 576 bp fragment covering the VPg region of TuMV‐GFP. The obtained products were Sanger sequenced (Genoscreen) and corresponding nucleotide and amino acid sequences were aligned to the VPg reference sequence of TuMV‐GFP UK1 (GenBank EF028235.1) by ClustalW using the BioEdit v. 7.2.5 software (Hall et al., 1999).

### 4.8 Statistical analysis

Kruskal–Wallis statistical tests were performed using the pgirmess package on R software (http://www.r‐project.org/).

## Supporting information


**FIGURE S1** Complementation of *eIF4E1* loss of function suppresses the enhanced susceptibility towards TuMV at 14 days postinoculation (dpi) (a) Phenotypic comparison of representative plants on TuMV‐GFP UK1 infection at 14 dpi. Photographs were taken under natural light conditions (left panel) and under wavelengths specific for chlorophyll excitation (middle panel) or green fluorescent protein (GFP) excitation (right panel) by using GFP Camera (PSI) fluorescence imaging. Fluorescence is represented by false colours ranging from blue (low intensity) to red (high intensity). Two independently obtained *eif4e1^KO^*;*eIF4E1* complemented lines (*eif4e1^KO^*;*eIF4E1‐1* and *eif4e1^KO^*;*eIF4E1‐2*) were used in the analyses. (b) Rosette fresh weight analysis of plants inoculated with TuMV‐GFP UK1 at 14 dpi. The aerial part was weighted for 12 TuMV‐GFP UK1‐inoculated plants of each genotype. Different letters depict significantly different groups identified by Kruskal–Wallis statistical tests at *p* < .05

**FIGURE S2** Complementation of *eIF4E1* loss‐of‐function suppresses the enhanced susceptibility towards TuMV at 21 days postinoculation (dpi). (a) Phenotypic comparison of representative plants on TuMV‐GFP UK1 infection at 21 dpi. Photographs were taken under natural light conditions (left panel) and under wavelengths specific for chlorophyll excitation (middle panel) or green fluorescent protein (GFP) excitation (right panel) by using GFP Camera (PSI) fluorescence imaging. Fluorescence is represented by false colours ranging from blue (low intensity) to red (high intensity). Two independently obtained *eif4e1^KO^*;*eIF4E1* complemented lines (*eif4e1^KO^*;*eIF4E1‐1* and *eif4e1^KO^*;*eIF4E1‐2*) were used in the analyses. (b) Rosette fresh weight analysis of plants inoculated with TuMV‐GFP UK1 at 21 dpi. The aerial part was weighed for 10 TuMV‐GFP UK1‐inoculated plants of each genotype. Different letters depict significantly different groups identified by Kruskal–Wallis statistical tests at *p* < .05

**FIGURE S3** Short‐exposure western blot revelations of Figure 4a and ImageJ quantifications of band intensity. (a) Western blot analysis on total protein extracts from mock‐inoculated wild‐type and TuMV‐GFP UK1‐inoculated plants at 21 days postinoculation (dpi). eIF4E1 and eIFiso4E protein accumulation was analysed by western blot on total plant protein extracts using specific antibodies. Equal loading was checked by western blot using anti‐actin antibodies. Each lane represents immunoblotted protein extracts obtained from a single independent plant. Images obtained with short‐exposure time. (b) Quantification of eIF4E1 accumulation levels using ImageJ software (imagej.nih.gov/ij). (c) Quantification of eIFiso4E levels using ImageJ software (imagej.nih.gov/ij). The surface value calculated for the eIFiso4E or eIF4E1 band was normalized to the surface value calculated for the actin band in the corresponding sample. Each barplot presents the normalized intensity of eIF4E1 or eIFiso4E bands in lanes shown in (a). Normalized eIF4E1 accumulation in *eifiso4e^KO^* (used as an internal control for eIF4E protein over‐accumulation) and normalized eIFiso4E accumulation in *eif4e1^KO^* are framed in red

**FIGURE S4** The functional *eIF4E1^N176K^* resistance allele does not trigger hypersusceptibility to TuMV at 21 days postinoculation (dpi). (a) Phenotypic comparison of representative plants on TuMV‐GFP UK1 infection at 21 dpi. Photographs were taken under natural light conditions (left panel) and under wavelengths specific for chlorophyll excitation (middle panel) or green fluorescent protein (GFP) excitation (right panel) by using GFP camera (PSI) fluorescence imaging. Fluorescence is represented by false colours ranging from blue (low intensity) to red (high intensity). Two independently obtained CRISPR‐induced *eIF4E1^N176K^* lines were used in the analyses. (b) Rosette fresh weight analysis of plants inoculated with TuMV‐GFP UK1 at 21 dpi. The aerial part was weighed for 24 wild‐type mock‐inoculated plants and at least 17 TuMV‐GFP UK1‐inoculated plants of each genotype. Different letters depict significantly different groups identified by Kruskal–Wallis statistical tests at *p* < .05

**FIGURE S5** Original western blot data. Images of the entire western blot membranes shown in Figure 4. Red rectangles delimit the images represented in Figure 4. The protein ladder (kDa) is shown on the left. Nonspecific proteins bands recognized by αAtActin and αAteIFiso4E antibodies are indicated

**TABLE S1** Recapitulative table of detected single nucleotide polymorphisms (SNPs) at the TuMV VPg coding sequence and the associated amino acid substitutions at relative to the reference TuMV‐GFP UK1 VPg sequence (GenBank EF028235.1) obtained from six wild‐type and 10 *eif4e1^KO^* TuMV‐GFP UK1‐inoculated plants. The identical polymorphisms corresponding to the amino acid substitutions N120G and S114L observed in TuMV VPg sequences obtained from both wild‐type and *eif4e1^KO^* plants are highlighted in green and blue, respectively

## Data Availability

Data available on request from the authors.

## References

[mpp13031-bib-0001] Abdelkefi, H. , Sugliani, M. , Ke, H. , Harchouni, S. , Soubigou‐Taconnat, L. , Citerne, S. et al. (2018) Guanosine tetraphosphate modulates salicylic acid signalling and the resistance of *Arabidopsis thaliana* to Turnip mosaic virus. Molecular Plant Pathology, 19, 634–646. 28220595 10.1111/mpp.12548 PMC6638062

[mpp13031-bib-0002] Agudelo‐Romero, P. , Carbonell, P. , Perez‐Amador, M.A. & Elena, S.F. (2008) Virus adaptation by manipulation of host’s gene expression. PLoS One, 3, e2397. 18545680 10.1371/journal.pone.0002397 PMC2398778

[mpp13031-bib-0003] Bart, R.S. & Taylor, N.J. (2017) New opportunities and challenges to engineer disease resistance in cassava, a staple food of African small‐holder farmers. PLoS Pathogens, 13, e1006287. 28493983 10.1371/journal.ppat.1006287 PMC5426740

[mpp13031-bib-0004] Bastet, A. , Lederer, B. , Giovinazzo, N. , Arnoux, X. , German‐Retana, S. , Reinbold, C. et al. (2018) Trans‐species synthetic gene design allows resistance pyramiding and broad‐spectrum engineering of virus resistance in plants. Plant Biotechnology Journal, 16, 1569–1581. 10.1111/pbi.12896 PMC6097130 29504210

[mpp13031-bib-0005] Bastet, A. , Robaglia, C. & Gallois, J.L. (2017) eIF4E resistance: natural variation should guide gene editing. Trends in Plant Science, 22, 411–419. 28258958 10.1016/j.tplants.2017.01.008

[mpp13031-bib-0006] Bastet, A. , Zafirov, D. , Giovinazzo, N. , Guyon‐Debast, A. , Nogué, F. , Robaglia, C. et al. (2019) Mimicking natural polymorphism in eIF4E by CRISPR‐Cas9 base editing is associated with resistance to potyviruses. Plant Biotechnology Journal, 17, 1736–1750. 30784179 10.1111/pbi.13096 PMC6686125

[mpp13031-bib-0007] Beauchemin, C. , Bougie, V. & Laliberté, J.F. (2005) Simultaneous production of two foreign proteins from a potyvirus‐based vector. Virus Research, 112, 1–8. 16022896 10.1016/j.virusres.2005.03.001

[mpp13031-bib-0008] Browning, K.S. & Bailey‐Serres, J. (2015) Mechanism of cytoplasmic mRNA translation. The Arabidopsis Book, 13, e0176. 26019692 10.1199/tab.0176 PMC4441251

[mpp13031-bib-0009] Caranta, C. (1997) Polygenic resistance of pepper to potyviruses consists of a combination of isolate‐specific and broad‐spectrum quantitative trait loci. Molecular Plant‐Microbe Interactions, 10, 872–878.

[mpp13031-bib-0010] Chandrasekaran, J. , Brumin, M. , Wolf, D. , Leibman, D. , Klap, C. , Pearlsman, M. et al. (2016) Development of broad virus resistance in non‐transgenic cucumber using CRISPR/Cas9 technology. Molecular Plant Pathology, 17, 1140–1153. 26808139 10.1111/mpp.12375 PMC6638350

[mpp13031-bib-0011] Cohn, M. , Bart, R.S. , Shybut, M. , Dahlbeck, D. , Gomez, M. , Morbitzer, R. et al. (2014) *Xanthomonas axonopodis* virulence is promoted by a transcription activator‐like effector – mediated induction of a SWEET sugar transporter in Cassava. Molecular Plant‐Microbe Interactions, 27, 1186–1198. 25083909 10.1094/MPMI-06-14-0161-R

[mpp13031-bib-0012] Combe, J.P. , Petracek, M.E. , Eldik, G.V. , Meulewaeter, F. & Twell, D. (2005) Translation initiation factors eIF4E and eIFiso4E are required for polysome formation and regulate plant growth in tobacco. Plant Molecular Biology, 57, 749–760. 15988567 10.1007/s11103-005-3098-x

[mpp13031-bib-0013] Cox, K.L. , Meng, F. , Wilkins, K.E. , Li, F. , Wang, P. , Booher, N.J. et al. (2017) TAL effector driven induction of a SWEET gene confers susceptibility to bacterial blight of cotton. Nature Communications, 8, 15588. 10.1038/ncomms15588 PMC5458083 28537271

[mpp13031-bib-0014] Duprat, A. , Caranta, C. , Revers, F. , Menand, B. , Browning, K.S. & Robaglia, C. (2002) The *Arabidopsis* eukaryotic initiation factor (iso)4E is dispensable for plant growth but required for susceptibility to potyviruses. The Plant Journal, 32, 927–934. 12492835 10.1046/j.1365-313x.2002.01481.x

[mpp13031-bib-0015] Estevan, J. , Maréna, A. , Callot, C. , Lacombe, S. , Moretti, A. , Caranta, C. et al. (2014) Specific requirement for translation initiation factor 4E or its isoform drives plant host susceptibility to tobacco etch virus. BMC Plant Biology, 14, 67. 24645730 10.1186/1471-2229-14-67 PMC3999954

[mpp13031-bib-0016] Gallois, J.L. , Charron, C. , Sanchez, F. , Pagny, G. , Houvenaghel, M.‐C. , Moretti, A. et al. (2010) Single amino acid changes in the turnip mosaic virus viral genome‐linked protein (VPg) confer virulence towards *Arabidopsis thaliana* mutants knocked out for eukaryotic initiation factors eIF(iso)4E and eIF(iso)4G. Journal of General Virology, 91, 288–293. 10.1099/vir.0.015321-0 19741065

[mpp13031-bib-0017] Gallois, J.L. , Moury, B. & German‐Retana, S. (2018) Role of the genetic background in resistance to plant viruses. International Journal of Molecular Sciences, 19, 2856. 10.3390/ijms19102856 PMC6213453 30241370

[mpp13031-bib-0018] Gauffier, C. , Lebaron, C. , Moretti, A. , Constant, C. , Moquet, F. , Bonnet, G. et al. (2016) A TILLING approach to generate broad‐spectrum resistance to potyviruses in tomato is hampered by *eIF4E* gene redundancy. The Plant Journal, 85, 717–729. 26850324 10.1111/tpj.13136

[mpp13031-bib-0019] Glais, L. , Faurez, F. , Tribodet, M. , Boulard, F. & Jacquot, E. (2015) The amino acid 419 in HC‐Pro is involved in the ability of PVY isolate N605 to induce necrotic symptoms on potato tubers. Virus Research, 208, 110–119. 26071382 10.1016/j.virusres.2015.05.024

[mpp13031-bib-0020] Gomez, M.A. , Lin, Z.D. , Moll, T. , Chauhan, R.D. , Hayden, L. , Renninger, K. et al. (2019) Simultaneous CRISPR/Cas9‐mediated editing of cassava eIF4E isoforms nCBP‐1 and nCBP‐2 reduces cassava brown streak disease symptom severity and incidence. Plant Biotechnology Journal, 17, 421–434. 30019807 10.1111/pbi.12987 PMC6335076

[mpp13031-bib-0021] Gonsalves, D. (1998) Control of papaya ringspot virus in papaya: a case study. Annual Review of Phytopathology, 36, 415–437. 10.1146/annurev.phyto.36.1.415 15012507

[mpp13031-bib-0051] Hall, T.A. (1999) BioEdit: A user‐friendly biological sequence alignment editor and analysis Pprogram for Windows 95/98/NT. Nucleic Acids Symposium Series, 41 95–98.

[mpp13031-bib-0022] Hosmillo, M. , Chaudhry, Y. , Kim, D.‐S. , Goodfellow, I. & Cho, K.‐O. (2014) Sapovirus translation requires an interaction between VPg and the cap binding protein eIF4E. Journal of Virology, 88, 12213–12221. 25142584 10.1128/JVI.01650-14 PMC4248917

[mpp13031-bib-0023] Jacob, P. , Avni, A. & Bendahmane, A. (2018) Translational research: exploring and creating genetic diversity. Trends in Plant Science, 23, 42–52. 29126790 10.1016/j.tplants.2017.10.002

[mpp13031-bib-0024] Kang, B.C. , Yeam, I. , Li, H. , Perez, K.W. & Jahn, M.M. (2007) Ectopic expression of a recessive resistance gene generates dominant potyvirus resistance in plants. Plant Biotechnology Journal, 5, 526–536. 17511813 10.1111/j.1467-7652.2007.00262.x

[mpp13031-bib-0026] Kusch, S. & Panstruga, R. (2017) *Mlo*‐based resistance: An apparently universal “weapon” to defeat powdery mildew disease. Molecular Plant‐Microbe Interactions, 30, 179–189. 28095124 10.1094/MPMI-12-16-0255-CR

[mpp13031-bib-0027] Lacombe, S. , Rougon‐Cardoso, A. , Sherwood, E. , Peeters, N. , Dahlbeck, D. , Van Esse, H.P. et al. (2010) Interfamily transfer of a plant pattern‐recognition receptor confers broad‐spectrum bacterial resistance. Nature Biotechnology, 28, 365–369. 10.1038/nbt.1613 20231819

[mpp13031-bib-0028] Lellis, A.D. , Allen, M.L. , Aertker, A.W. , Tran, J.K. , Hillis, D.M. , Harbin, C.R. et al. (2010) Deletion of the eIFiso4G subunit of the *Arabidopsis* eIFiso4F translation initiation complex impairs health and viability. Plant Molecular Biology, 74, 249–263. 20694742 10.1007/s11103-010-9670-z PMC2938417

[mpp13031-bib-0029] Li, W. , Deng, Y. , Ning, Y. , He, Z. & Wang, G.‐L. (2020) Exploiting broad‐spectrum disease resistance in crops: from molecular dissection to breeding. Annual Review of Plant Biology, 71, 575–603. 10.1146/annurev-arplant-010720-022215 32197052

[mpp13031-bib-0030] Mäkinen, K. (2020) Plant susceptibility genes as a source for potyvirus resistance. Annals of Applied Biology, 176, 122–129.

[mpp13031-bib-0031] Mayberry, L.K. , Allen, M.L. , Nitka, K.R. , Campbell, L. , Murphy, P.A. & Browning, K.S. (2011) Plant cap‐binding complexes eukaryotic initiation factors eIF4F and eIFiso4F: molecular specificity of subunit binding. Journal of Biological Chemistry, 286, 42566–42574. 10.1074/jbc.M111.280099 PMC3234931 21965660

[mpp13031-bib-0032] Michel, V. , Julio, E. , Candresse, T. , Cotucheau, J. , Decorps, C. , Volpatti, R. et al. (2019) A complex *eIF4E* locus impacts the durability of *va* resistance to potato virus Y in tobacco. Molecular Plant Pathology, 20, 1051–1066. 31115167 10.1111/mpp.12810 PMC6640182

[mpp13031-bib-0033] Moury, B. , Palloix, A. , Caranta, C. , Gognalons, P. , Souche, S. , Selassie, K.G. et al. (2005) Serological, molecular, and pathotype diversity of *Pepper veinal mottle virus* and *Chili veinal mottle virus* . Phytopathology, 95, 227–232. 18943114 10.1094/PHYTO-95-0227

[mpp13031-bib-0034] Mushtaq, M. , Sakina, A. , Wani, S.H. , Shikari, A.B. , Tripathi, P. , Zaid, A. et al. (2019) Harnessing genome editing techniques to engineer disease resistance in plants. Frontiers in Plant Science, 10, 550. 31134108 10.3389/fpls.2019.00550 PMC6514154

[mpp13031-bib-0035] Nicaise, V. , Gallois, J.L. , Chafiai, F. , Allen, L.M. , Schurdi‐Levraud, V. , Browning, K.S. et al. (2007) Coordinated and selective recruitment of eIF4E and eIF4G factors for potyvirus infection in *Arabidopsis thaliana* . FEBS Letters, 581, 1041–1046. 17316629 10.1016/j.febslet.2007.02.007

[mpp13031-bib-0036] Oliva, R. , Ji, C. , Atienza‐Grande, G. , Huguet‐Tapia, J.C. , Perez‐Quintero, A. , Li, T. et al. (2019) Broad‐spectrum resistance to bacterial blight in rice using genome editing. Nature Biotechnology, 37, 1344–1350. 10.1038/s41587-019-0267-z PMC6831514 31659337

[mpp13031-bib-0037] Pružinská, A. , Tanner, G. , Aubry, S. , Anders, I. , Moser, S. , Müller, T. et al. (2005) Chlorophyll breakdown in senescent *Arabidopsis* leaves. Characterization of chlorophyll catabolites and of chlorophyll catabolic enzymes involved in the degreening reaction. Plant Physiology, 139, 52–63. 16113212 10.1104/pp.105.065870 PMC1203357

[mpp13031-bib-0038] Pyott, D.E. , Fei, Y. & Molnar, A. (2020) Potential for gene editing in antiviral resistance. Current Opinion in Virology, 42, 47–52. 32512313 10.1016/j.coviro.2020.04.005

[mpp13031-bib-0039] Quenouille, J. , Paulhiac, E. , Moury, B. & Palloix, A. (2014) Quantitative trait loci from the host genetic background modulate the durability of a resistance gene: a rational basis for sustainable resistance breeding in plants. Heredity, 112, 579–587. 24569635 10.1038/hdy.2013.138 PMC4023440

[mpp13031-bib-0040] Quenouille, J. , Saint‐Felix, L. , Moury, B. & Palloix, A. (2016) Diversity of genetic backgrounds modulating the durability of a major resistance gene. Analysis of a core collection of pepper landraces resistant to *Potato virus Y* . Molecular Plant Pathology, 17, 296–302. 25967744 10.1111/mpp.12277 PMC6638519

[mpp13031-bib-0041] Robaglia, C. & Caranta, C. (2006) Translation initiation factors: a weak link in plant RNA virus infection. Trends in Plant Science, 11, 40–45. 16343979 10.1016/j.tplants.2005.11.004

[mpp13031-bib-0042] Rodriguez‐Moreno, L. , Song, Y. & Thomma, B.P. (2017) Transfer and engineering of immune receptors to improve recognition capacities in crops. Current Opinion in Plant Biology, 38, 42–49. 28472757 10.1016/j.pbi.2017.04.010

[mpp13031-bib-0043] Rubio, M. , Caranta, C. & Palloix, A. (2008) Functional markers for selection of potyvirus resistance alleles at the *pvr2*‐*eIF4E* locus in pepper using tetra‐primer ARMS‐PCR. Genome, 51, 767–771. 18772955 10.1139/G08-056

[mpp13031-bib-0044] Ruffel, S. , Dussault, M.H. , Palloix, A. , Moury, B. , Bendahmane, A. , Robaglia, C. et al. (2002) A natural recessive resistance gene against potato virus Y in pepper corresponds to the eukaryotic initiation factor 4E (elF4E). The Plant Journal, 32, 1067–1075. 12492847 10.1046/j.1365-313x.2002.01499.x

[mpp13031-bib-0045] Sanchez, F. , Manrique, P. , Mansilla, C. , Lunello, P. , Wang, X. , Rodrigo, G. et al. (2015) Viral strain‐specific differential alterations in *Arabidopsis* developmental patterns. Molecular Plant‐Microbe Interactions, 28, 1304–1315. 26646245 10.1094/MPMI-05-15-0111-R

[mpp13031-bib-0046] Sato, M. , Nakahara, K. , Yoshii, M. , Ishikawa, M. & Uyeda, I. (2005) Selective involvement of members of the eukaryotic initiation factor 4E family in the infection of *Arabidopsis thaliana* by potyviruses. FEBS Letters, 579, 1167–1171. 15710407 10.1016/j.febslet.2004.12.086

[mpp13031-bib-0047] Schmitt‐Keichinger, C. (2019) Manipulating cellular factors to combat viruses: a case study from the plant eukaryotic translation initiation factors eIF4. Frontiers in Microbiology, 10, 17. 30804892 10.3389/fmicb.2019.00017 PMC6370628

[mpp13031-bib-0049] Veillet, F. , Durand, M. , Kroj, T. , Cesari, S. & Gallois, J.‐L. (2020) Precision breeding made real with CRISPR: illustration through genetic resistance to pathogens. Plant Communications, 1, 100102. 33367260 10.1016/j.xplc.2020.100102 PMC7747970

[mpp13031-bib-0050] Wang, A. & Krishnaswamy, S. (2012) Eukaryotic translation initiation factor 4E‐mediated recessive resistance to plant viruses and its utility in crop improvement. Molecular Plant Pathology, 13, 795–803. 22379950 10.1111/j.1364-3703.2012.00791.x PMC6638641

[mpp13031-bib-0048] van Wersch, S. , Tian, L. , Hoy, R. & Li, X. (2020) Plant NLRs: the whistleblowers of plant immunity. Plant Communications, 1, 100016. 33404540 10.1016/j.xplc.2019.100016 PMC7747998

