# Supplementary figures and images for: When a knockout is an Achilles’ heel: Resistance to one potyvirus species triggers hypersusceptibility to another one in Arabidopsis thaliana
Source: Mol Plant Pathol. 2020 Dec 29;22(3):334–47. doi: 10.1111/mpp.13031 (PMC7865081; doi:10.1111/mpp.13031)

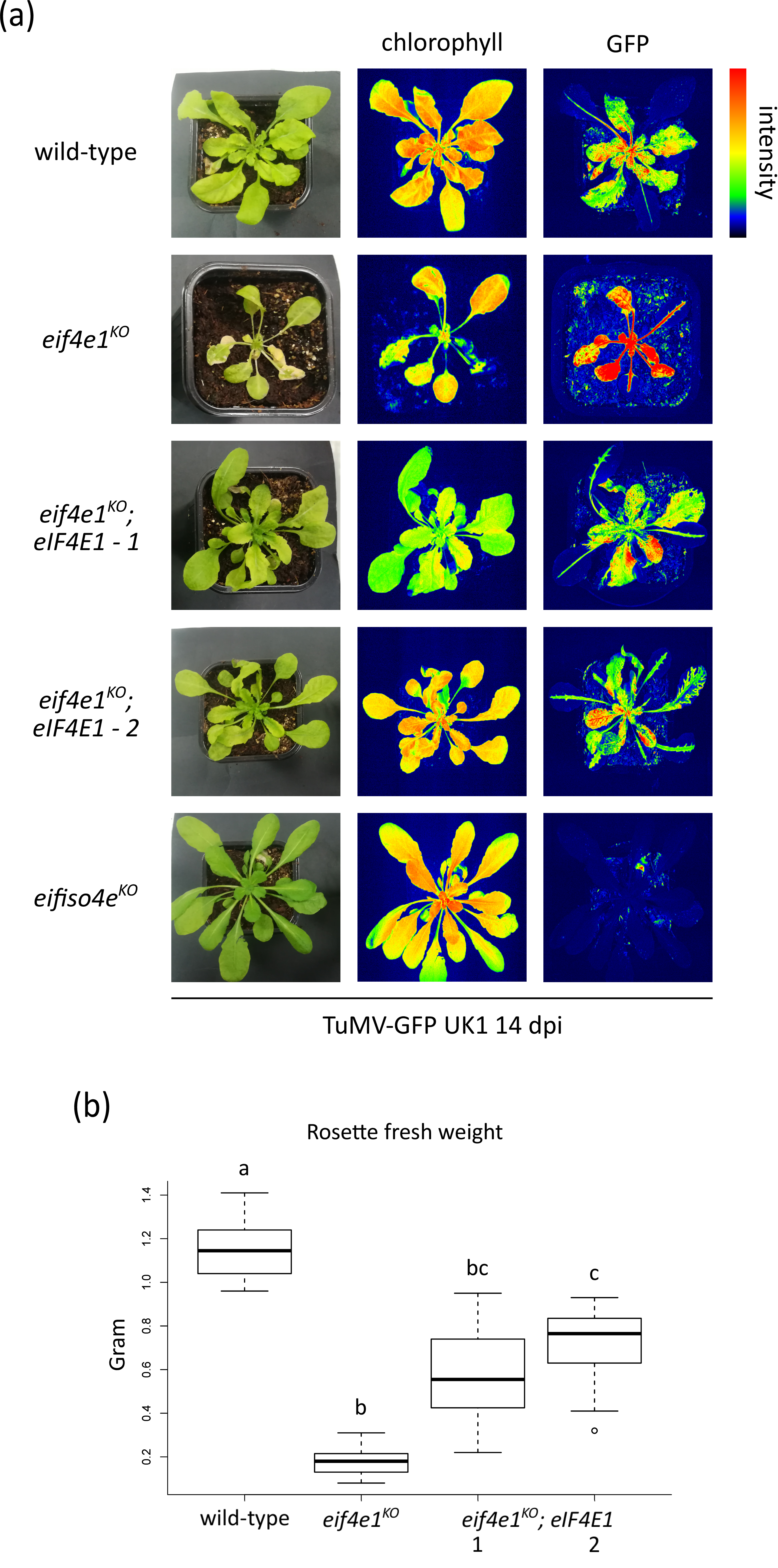

Supplement: Supplementary file 1 — FIGURE S1 Complementation of eIF4E1 loss of function suppresses the enhanced susceptibility towards TuMV at 14 days postinoculation (dpi) (a) Phenotypic comparison of representative plants on TuMV‐GFP UK1 infection at 14 dpi. Photographs were taken under natural light conditions (left panel) and under wavelengths specific for chlorophyll excitation (middle panel) or green fluorescent protein (GFP) excitation (right panel) by using GFP Camera (PSI) fluorescence imaging. Fluorescence is represented by false colours ranging from blue (low intensity) to red (high intensity). Two independently obtained eif4e1KO;eIF4E1 complemented lines (eif4e1KO;eIF4E1‐1 and eif4e1KO;eIF4E1‐2) were used in the analyses. (b) Rosette fresh weight analysis of plants inoculated with TuMV‐GFP UK1 at 14 dpi. The aerial part was weighted for 12 TuMV‐GFP UK1‐inoculated plants of each genotype. Different letters depict significantly different groups identified by Kruskal–Wallis statistical tests at p < .05 [file MPP-22-334-s001.tif]

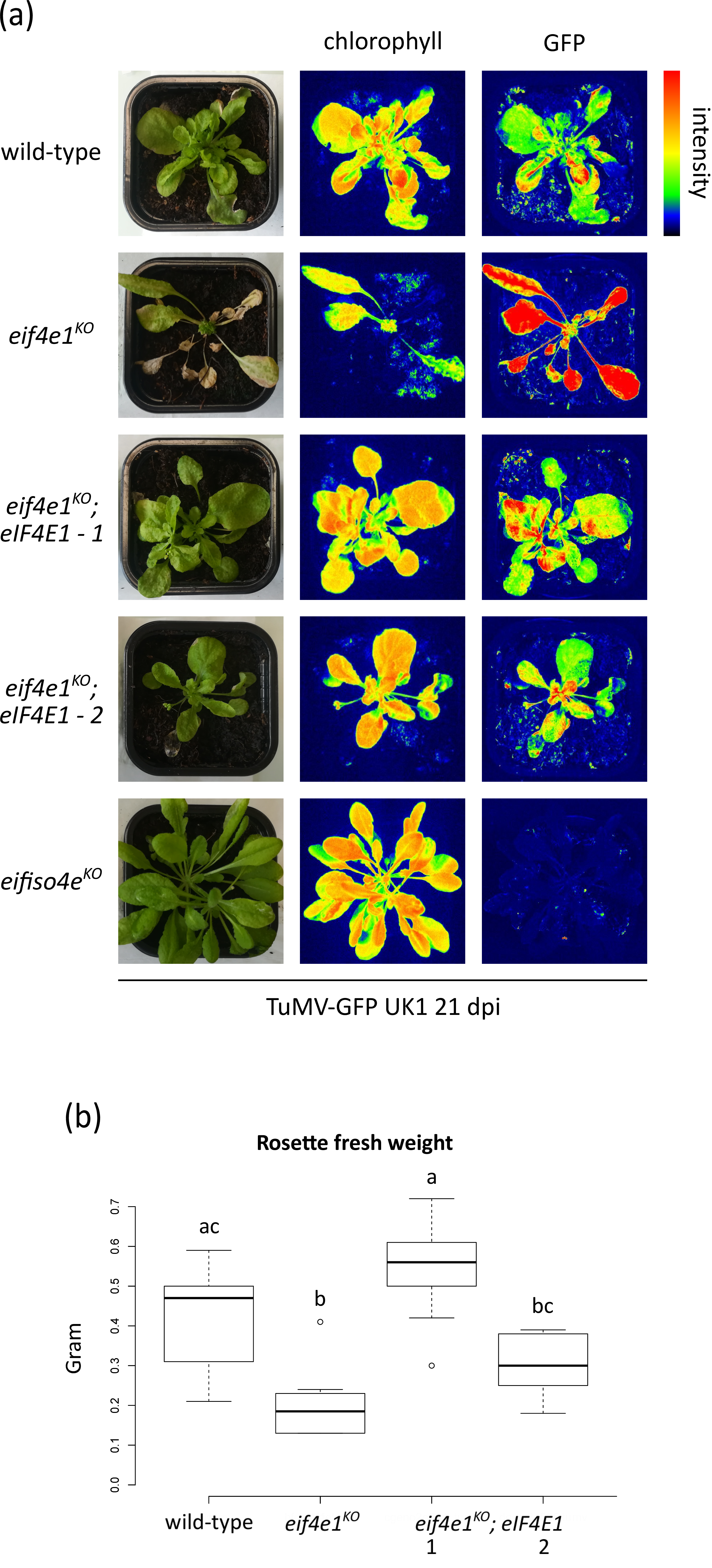

Supplement: Supplementary file 2 — FIGURE S2 Complementation of eIF4E1 loss‐of‐function suppresses the enhanced susceptibility towards TuMV at 21 days postinoculation (dpi). (a) Phenotypic comparison of representative plants on TuMV‐GFP UK1 infection at 21 dpi. Photographs were taken under natural light conditions (left panel) and under wavelengths specific for chlorophyll excitation (middle panel) or green fluorescent protein (GFP) excitation (right panel) by using GFP Camera (PSI) fluorescence imaging. Fluorescence is represented by false colours ranging from blue (low intensity) to red (high intensity). Two independently obtained eif4e1KO;eIF4E1 complemented lines (eif4e1KO;eIF4E1‐1 and eif4e1KO;eIF4E1‐2) were used in the analyses. (b) Rosette fresh weight analysis of plants inoculated with TuMV‐GFP UK1 at 21 dpi. The aerial part was weighed for 10 TuMV‐GFP UK1‐inoculated plants of each genotype. Different letters depict significantly different groups identified by Kruskal–Wallis statistical tests at p < .05 [file MPP-22-334-s002.tif]

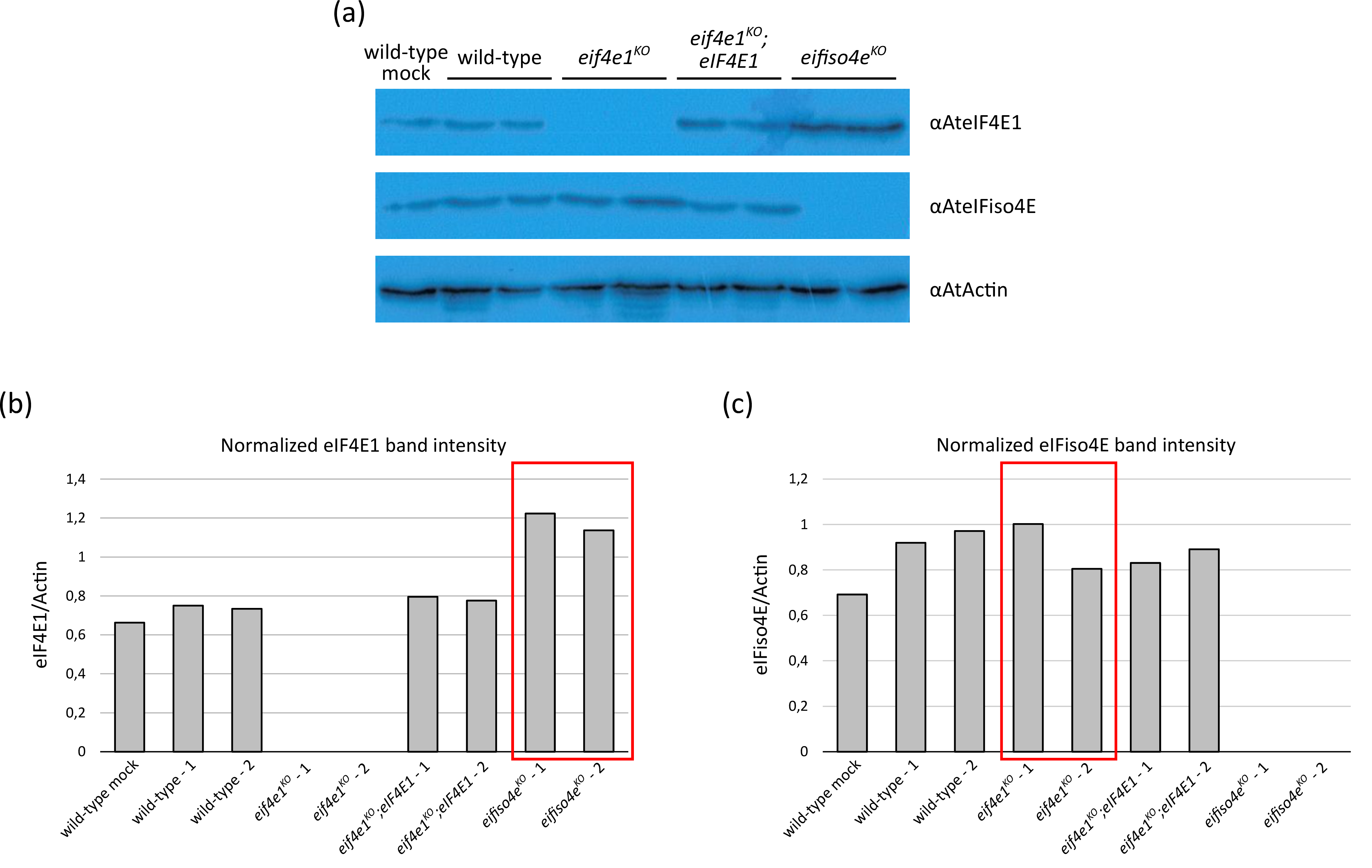

Supplement: Supplementary file 3 — FIGURE S3 Short‐exposure western blot revelations of Figure 4a and ImageJ quantifications of band intensity. (a) Western blot analysis on total protein extracts from mock‐inoculated wild‐type and TuMV‐GFP UK1‐inoculated plants at 21 days postinoculation (dpi). eIF4E1 and eIFiso4E protein accumulation was analysed by western blot on total plant protein extracts using specific antibodies. Equal loading was checked by western blot using anti‐actin antibodies. Each lane represents immunoblotted protein extracts obtained from a single independent plant. Images obtained with short‐exposure time. (b) Quantification of eIF4E1 accumulation levels using ImageJ software (imagej.nih.gov/ij). (c) Quantification of eIFiso4E levels using ImageJ software (imagej.nih.gov/ij). The surface value calculated for the eIFiso4E or eIF4E1 band was normalized to the surface value calculated for the actin band in the corresponding sample. Each barplot presents the normalized intensity of eIF4E1 or eIFiso4E bands in lanes shown in (a). Normalized eIF4E1 accumulation in eifiso4eKO (used as an internal control for eIF4E protein over‐accumulation) and normalized eIFiso4E accumulation in eif4e1KO are framed in red [file MPP-22-334-s003.tif]

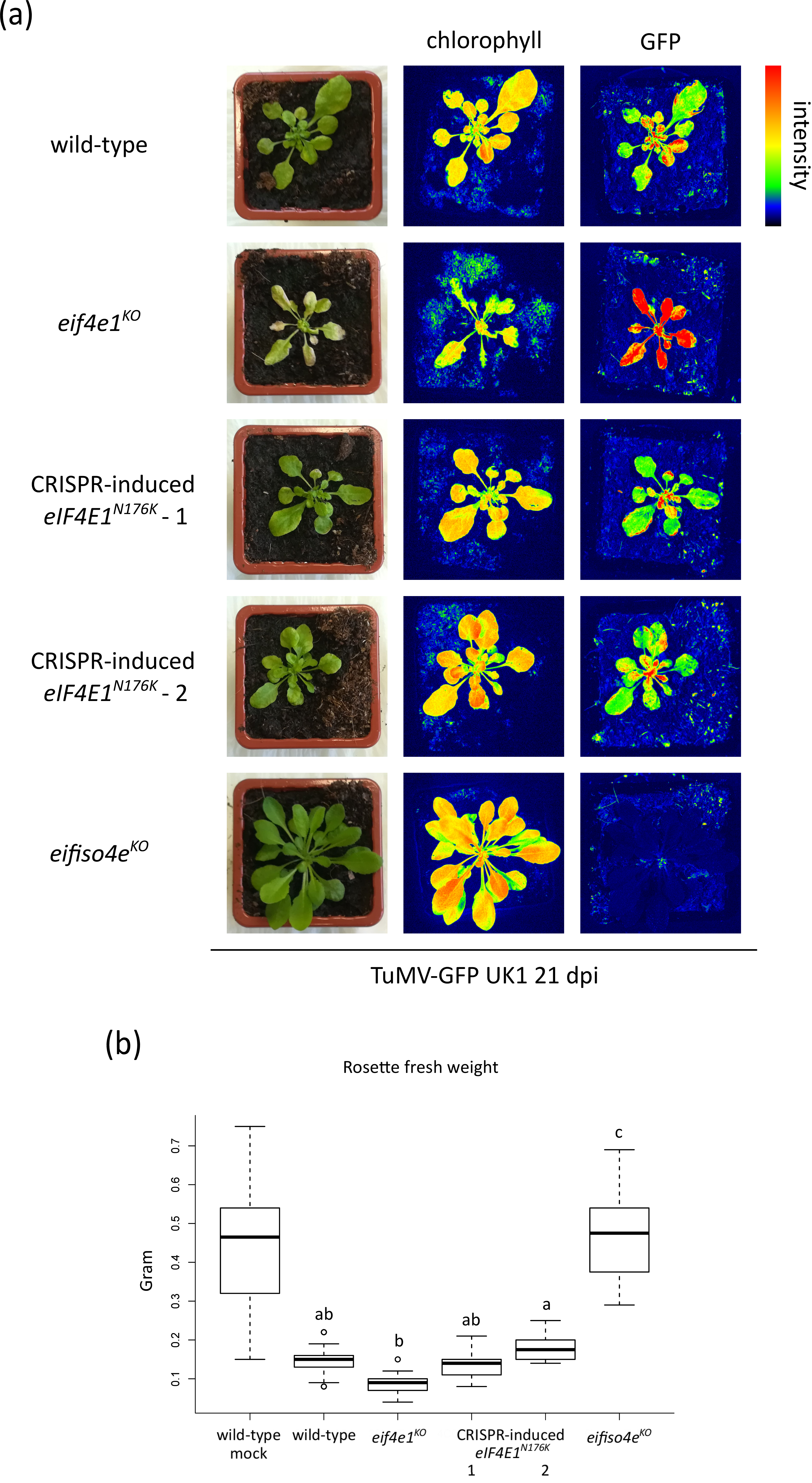

Supplement: Supplementary file 4 — FIGURE S4 The functional eIF4E1N176K resistance allele does not trigger hypersusceptibility to TuMV at 21 days postinoculation (dpi). (a) Phenotypic comparison of representative plants on TuMV‐GFP UK1 infection at 21 dpi. Photographs were taken under natural light conditions (left panel) and under wavelengths specific for chlorophyll excitation (middle panel) or green fluorescent protein (GFP) excitation (right panel) by using GFP camera (PSI) fluorescence imaging. Fluorescence is represented by false colours ranging from blue (low intensity) to red (high intensity). Two independently obtained CRISPR‐induced eIF4E1N176K lines were used in the analyses. (b) Rosette fresh weight analysis of plants inoculated with TuMV‐GFP UK1 at 21 dpi. The aerial part was weighed for 24 wild‐type mock‐inoculated plants and at least 17 TuMV‐GFP UK1‐inoculated plants of each genotype. Different letters depict significantly different groups identified by Kruskal–Wallis statistical tests at p < .05 [file MPP-22-334-s004.tif]

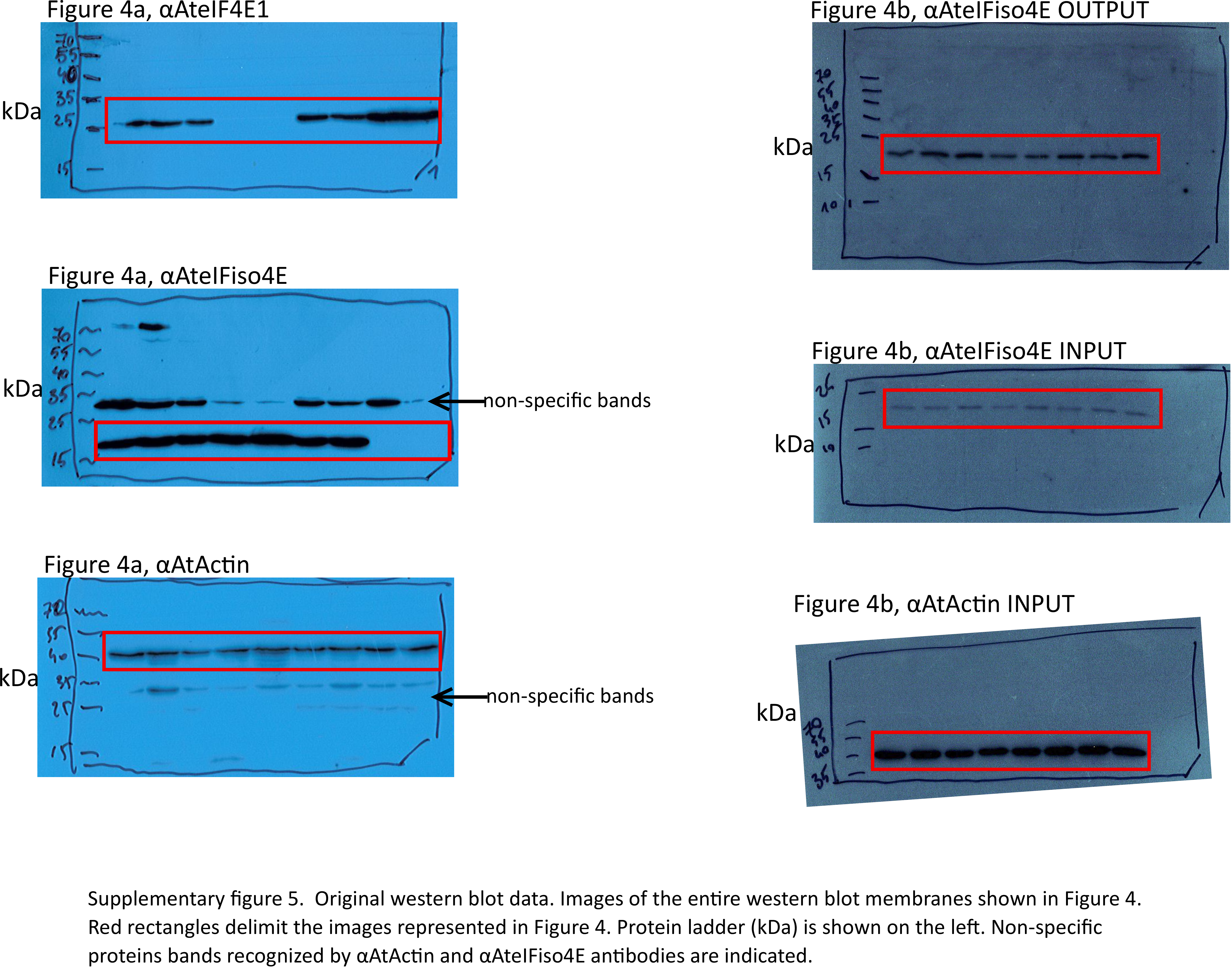

Supplement: Supplementary file 5 — FIGURE S5 Original western blot data. Images of the entire western blot membranes shown in Figure 4. Red rectangles delimit the images represented in Figure 4. The protein ladder (kDa) is shown on the left. Nonspecific proteins bands recognized by αAtActin and αAteIFiso4E antibodies are indicated [file MPP-22-334-s005.tif]
