# Supplementary material for: When a knockout is an Achilles’ heel: Resistance to one potyvirus species triggers hypersusceptibility to another one in Arabidopsis thaliana
Source: Mol Plant Pathol. 2020 Dec 29;22(3):334–47. doi: 10.1111/mpp.13031 (PMC7865081; doi:10.1111/mpp.13031)
Supplement: Supplementary file 6 — TABLE S1 Recapitulative table of detected single nucleotide polymorphisms (SNPs) at the TuMV VPg coding sequence and the associated amino acid substitutions at relative to the reference TuMV‐GFP UK1 VPg sequence (GenBank EF028235.1) obtained from six wild‐type and 10 eif4e1KO TuMV‐GFP UK1‐inoculated plants. The identical polymorphisms corresponding to the amino acid substitutions N120G and S114L observed in TuMV VPg sequences obtained from both wild‐type and eif4e1KO plants are highlighted in green and blue, respectively [file MPP-22-334-s006.pdf]

Sample	Nucleotide polymorphism			Amino acid polymorphism		
	Reference VPg	SNP	Position	Reference VPg	AA substitution	Position
wild-type 1	A	R (A or G)	338	D (GAC)	G (GGC)	113
wild-type 2						
wild-type 3						
wild-type 4						
wild-type 5	A	R (A or G)	358	N (AAU)	D (GAU)	120
wild-type 6	C	Y (C or U)	341	S (UCA)	L (UUA)	114
eif4e1^{KO} 1						
eif4e1^{KO} 2						
eif4e1^{KO} 3						
eif4e1^{KO} 4						
eif4e1^{KO} 5						
eif4e1^{KO} 6	A	R (A or G)	358	N (AAU)	D (GAU)	120
eif4e1^{KO} 7	A	R (A or G)	310	M (AUG)	V (GUG)	104
eif4e1^{KO} 8						
eif4e1^{KO} 9	C	Y (C or U)	341	S (UCA)	L (UUA)	114
eif4e1^{KO} 10	A	M (A or C)	344	N (AAU)	T (ACU)	115

Supplementary table 1. Recapitulative table of detected single nucleotide polymorphisms (SNP) at the TuMV VPg coding sequence and the associated amino acid substitutions at relative to the reference TuMV-GFP UK1 VPg sequence (GenBank ID: EF028235.1) obtained from six wild-type and ten *eif4e1*^{KO} TuMV-GFP UK1-inoculated plants. The identical polymorphisms corresponding to the amino acid substitutions N120G and S114L observed in TuMV VPg sequences obtained from both wild-type and *eif4e1*^{KO} plants are highlighted in green and blue, respectively.
